# Building genetic healthcare together: an Australian co-production three-phase mixed-methods research protocol with people with intellectual disability

**DOI:** 10.1136/bmjopen-2025-110086

**Published:** 2026-07-10

**Authors:** Iva Strnadová, Michelle Tso, Julie Loblinzk Refalo, Natalie Roberts, Joanne Danker, Skie Sarfaraz, Jackie Boyle, Bronwyn Terrill, Celia Halliburton, Claudia Pantoja Mardones, Sarah Hayes, Sam Hurd, Caitlin King, Kristine Barlow-Stewart, Elizabeth Evans, Helen Leonard, Helen Mar Fan, Jonathan Rodgers, Yvette Vella, Julie McGaughran, Stephanie Best, Erin Turbitt, Jackie Leach Scully, Greg Pratt, Elizabeth E Palmer

**Affiliations:** 1 School of Education, University of New South Wales, Sydney, New South Wales, Australia; 2 University of New South Wales Disability Innovation Institute, Sydney, New South Wales, Australia; 3 Self Advocacy Sydney, Sydney, New South Wales, Australia; 4 Discipline of Paediatrics and Child Health, School of Clinical Medicine, Faculty of Medicine and Health, University of New South Wales, Sydney, New South Wales, Australia; 5 Genetics of Learning Disability Service, Waratah, New South Wales, Australia; 6 St Vincent’s Healthcare Clinical Campus, School of Clinical Medicine, Faculty of Medicine and Health, University of New South Wales, Sydney, New South Wales, Australia; 7 Centre for Population Genomics, Garvan Institute of Medical Research, Darlinghurst, New South Wales, Australia; 8 Australian Genomics, Melbourne, Victoria, Australia; 9 NSW Ministry of Health, St Leonards, New South Wales, Australia; 10 Central Queensland University, Brisbane, Queensland, Australia; 11 Northern Clinical School, Faculty of Medicine and Health, University of Sydney, St Leonards, New South Wales, Australia; 12 Psychologist in private practice, Sydney, New South Wales, Australia; 13 The University of Western Australia, Perth, Western Australia, Australia; 14 The Kids Research Institute Australia, Nedlands, Western Australia, Australia; 15 Genetic Health Queensland, Brisbane, Queensland, Australia; 16 Primary and Community Health, Northern Sydney Local Health District, St Leonards, New South Wales, Australia; 17 School of Medicine, The University of Queensland, Brisbane, Queensland, Australia; 18 University of Melbourne, Melbourne, Victoria, Australia; 19 University of Technology Sydney, Sydney, New South Wales, Australia; 20 Centre for Clinical Genetics, Sydney Children’s Hospitals Network, Sydney, New South Wales, Australia

**Keywords:** Health Equity, GENETICS, MEDICAL EDUCATION & TRAINING, Developmental neurology & neurodisability, Australian Aboriginal and Torres Strait Islander Peoples, Health Literacy

## Abstract

**Introduction:**

People with intellectual disability face significant barriers to accessing genetic healthcare, including during appointments. Many adults with intellectual disability are denied opportunities to discuss genetic testing that could improve their health and well-being. This study follows best practice in inclusive research, with co-researchers with intellectual disability co-leading alongside educational, disability and clinical experts to co-design and implement a respectful, accessible and inclusive national model of genetic healthcare.

**Methods and analysis:**

This three-phase qualitative study positions people with intellectual disability as active researchers and partners throughout. Phase 1 will identify barriers and enablers to genetic healthcare for people with intellectual disability by engaging with approximately 100 participants—people with intellectual disability (with and without genetic testing experience), families/support people and genetic healthcare professionals—through interviews, focus groups, arts-based methods and yarning circles. Phase 2 will co-design and evaluate Guiding Principles, providing solutions to identified barriers and leveraging identified facilitators. Phase 3 will develop practical resources using workshops across Australia, guided by three Community Engagement Groups, including people with intellectual disability, families/support people and healthcare providers. This phase will also investigate strategies to optimise the national roll-out and implementation of resources among health professionals, people with intellectual disability and their families/carers. Aboriginal and Torres Strait Islander research methods are integrated throughout, led by Indigenous researchers.

**Ethics and dissemination:**

Ethics approval has been granted by UNSW Sydney Human Research Ethics Committee (HC230353) and the Australian Institute of Aboriginal and Torres Strait Islander Studies (REC-0284). Participants will provide written consent using Easy Read or plain English materials. Findings will be disseminated through journal articles, conferences, websites and accessible formats, including Easy Read summaries and videos.

See the Graphical Abstract for a visual summary in online supplemental file 1

**STRENGTHS AND LIMITATIONS OF THIS STUDY**

- Authentic, inclusive research methodology with people with intellectual disability as co-researchers and co-leaders throughout all phases.
- Multimodal data collection methods (interviews, focus groups and arts-based approaches), including Aboriginal and Torres Strait Islander research methodologies (yarning circles) with Indigenous researcher leadership.
- A sequential three-phase design ensures participant involvement from research conception through to national implementation planning.
- A convenience sampling approach may limit the generalisability of findings despite efforts to recruit across age, geography, support needs and genetic testing experience.
- Resource constraints may limit the depth of engagement possible in remote areas or with specific population subgroups.

## Introduction

Intellectual disability refers to neurodevelopmental conditions that begin in childhood, impacting conceptual, social and practical areas of living.^1^ However, each person’s specific strengths and support needs are unique. Traditional healthcare typically emphasises limitations rather than capabilities, often excluding people with intellectual disability from decision-making processes.^2^ There is compelling evidence that many health professionals exclude people with intellectual disability from consultations, misattribute health problems to their disability (known as diagnostic overshadowing) and sometimes display unprofessional behaviour.^3 4^ The right to equitable and accessible healthcare, as mandated by the *United Nations Convention on the Rights of Persons with Disabilities (2006*),^5^ is being ignored.

Healthcare workers lack training and confidence in providing healthcare to people with intellectual disability and tend to make assumptions about the capacity of the person to make their own informed choices.^2^ They often direct communications to family members, assuming that the person with intellectual disability cannot understand or contribute to healthcare discussions.^6 7^ Our work challenges these assumptions by recognising that with appropriate accommodations and respectful engagement, people with intellectual disability can and should be active participants in their healthcare decisions.^8^

People with intellectual disability, especially those with high support needs—that is, people who require extensive or pervasive support across most areas of daily living, which includes many people with severe or profound intellectual disability and/or multiple disabilities—experience immense health inequalities, including premature mortality and disproportionate rates of potentially avoidable deaths as a result of late diagnosis or mismanagement.^9–12^ People with intellectual disability are frequently neglected and abused within the healthcare system.^13 14^ There is growing awareness of the need for strengths-based, person-centred and trauma-informed healthcare that promotes safety, trust, collaboration, choice and empowerment to ensure that people with intellectual disability are engaged in their own healthcare.^15^

Genetic healthcare is the application of genetic knowledge in diagnosis, prevention and treatment to improve health outcomes.^16 17^ Genetic studies have revolutionised our understanding of the causes of intellectual disability, with over 1000 genes now implicated.^18 19^ Chromosomal microarray, screening for fragile X syndrome and then more comprehensive genomic testing, such as exome or genome sequencing, are recommended as diagnostic tests for neurodevelopmental conditions.^19 20^ Intellectual disability and/or global developmental delay are the most frequent reasons for medical referral, accounting for 20%–30% of genetic testing across all ages and 50%–70% in paediatric populations.^16 17 21–23^ Identifying the genetic basis of a person’s intellectual disability has numerous potential benefits. Clinically, it enables surveillance for known complications, guides medical referrals and informs specific therapies. Individually, it helps people understand their condition, connects them with peer support groups, improves access to appropriate services, informs reproductive choices and contributes to better quality-of-life outcomes.^18 24–27^

However, whether people with intellectual disability are fully benefiting from these potential advantages has historically been under-researched. Our recent systematic literature review found that only seven studies had been conducted up to 2021 to investigate the opinions and experiences of people with intellectual disability in genetic counselling and testing, highlighting a major gap in knowledge.^28^ We also found that few resources on genetics and genetic conditions had been developed specifically for people with intellectual disability, further excluding this group from informed participation in genetic healthcare.^28 29^

The lack of appropriate resources and services is likely to have a disproportionate impact on Australian Aboriginal and Torres Strait Islander people with intellectual disability. The prevalence of intellectual disability in this population is two to three times higher than among non-Indigenous Australians.^30 31^ Beyond the common challenges faced by people with intellectual disability, they also encounter layers of institutional and personal discrimination,^32^ use general and genetic health services less frequently than non-Indigenous people and consistently report being treated in excluding or dismissive ways by healthcare professionals.^13^ Strategies to realise the promise of genetic healthcare must address these issues through the development and implementation of culturally appropriate models of genetic healthcare for Aboriginal and Torres Strait Islander people with intellectual disability. This research must be led by Indigenous researchers in partnership with Indigenous people, families, professionals and communities.

Our initial Australian pilot study interviewed adults with intellectual disability who had previous contact with genetics services.^28^ The findings painted a concerning picture: participants often reported feeling excluded and unsupported, experiencing anxiety and depression as a result. We identified numerous missed opportunities for genetic diagnoses that could have informed practical healthcare plans, disability supports and therapies. The study highlighted the urgent need for new approaches and for expanding our research to include people with higher support needs, those from different regions, family members, support people and clinicians themselves.^8 28^

## Project aims

The GeneEQUAL research programme aims to develop and evaluate an inclusive, person-centred and respectful model of genetic healthcare for people with intellectual disability that addresses the limitations and inequities in the current provision. Our three main aims are:

1. *To understand what prevents and enables access to and provision of inclusive genetic healthcare*. We aim to identify barriers and enablers from the perspectives of people with intellectual disability across all support needs, including Aboriginal and Torres Strait Islander people, their families and support people and healthcare professionals.
2. *To co-design evidence-based solutions*. We aim to develop Guiding Principles that healthcare services can use to overcome barriers and create inclusive, person-centred and respectful genetic healthcare for all people with intellectual disability.
3. *To create resources for real-world implementation*. We aim to co-produce a suite of resources and implementation strategies that enable services to deliver best practices in genetic healthcare nationally and globally.

Across these aims, we are committed to addressing the challenges faced by Aboriginal and Torres Strait Islander people with intellectual disability, ensuring our approach is culturally safe and appropriate.

An Easy Read summary of this article is available in the online supplemental material.

10.1136/bmjopen-2025-110086.supp2 Supplementary data

## Methods and analysis

### Theoretical framework and inclusive design

This project combines social constructivist epistemology and critical realist ontology to support meaningful co-production with people with intellectual disability.^33^ Our approach recognises that while genetic conditions have a biological basis,^34 35^ their impact is shaped by social and cultural factors.^35 36^ The centring of co-production aligns with our commitment to doing research *with* people with intellectual disability rather than *on* them.

**Figure 1:**
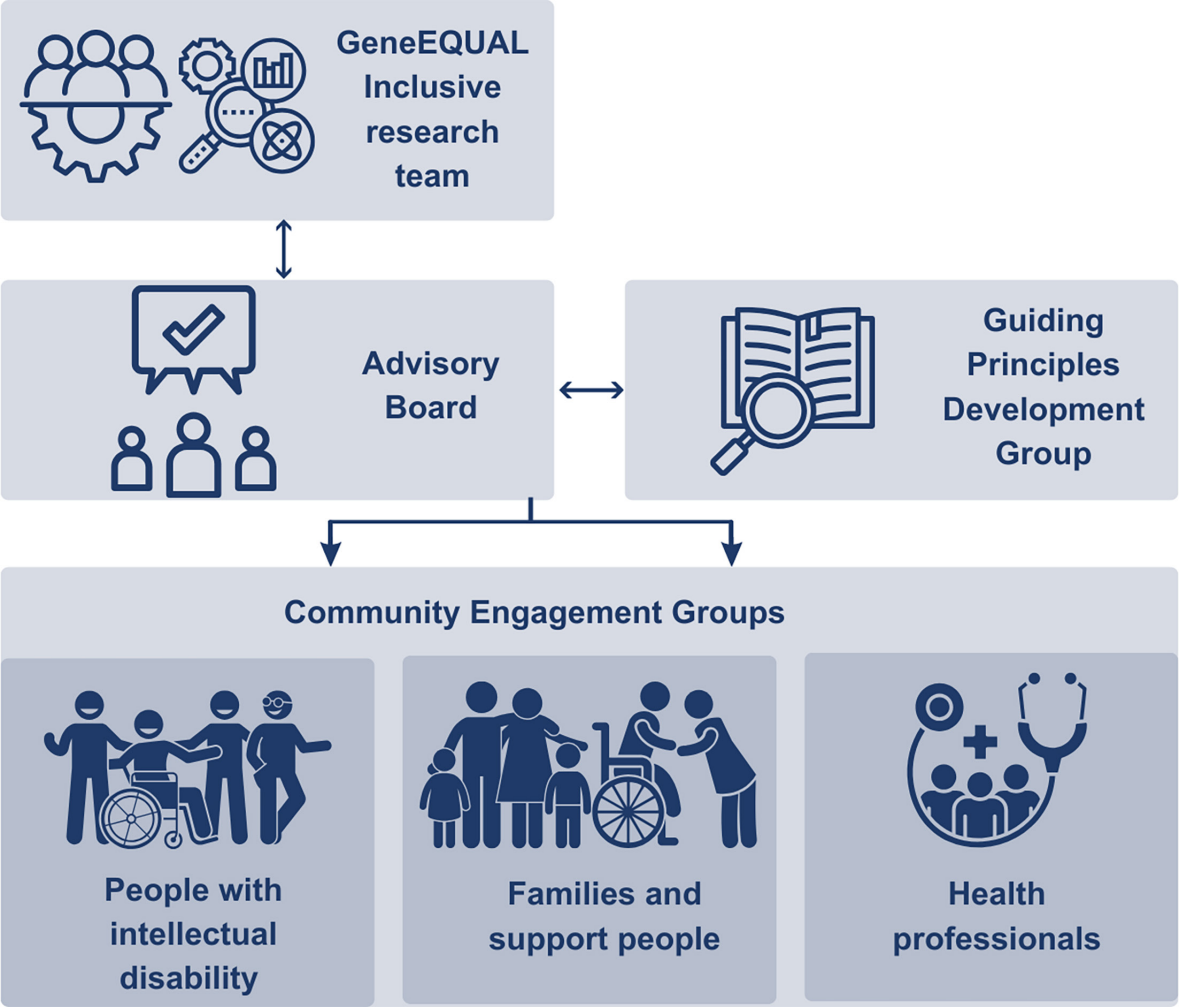
Project engagement structure.

The study adheres to the principles described in ‘Doing Research Inclusively: Co-Production in Action’,^37^ ensuring all stakeholders have meaningful roles in knowledge creation. As shown in figure 1, the three Community Engagement Groups (CEGs) are organised by stakeholder type: (1) people with intellectual disability, (2) families and support people and (3) health professionals. This structure was chosen to ensure that each group can discuss issues relevant to their specific perspectives and experiences without the potential power imbalances that can arise in mixed stakeholder groups. In particular, separating the group of people with intellectual disability from healthcare professionals and family members ensures that their voices are not overshadowed and that they can speak freely about their experiences. All three CEGs contribute to the same research phases, and their perspectives are brought together during Advisory Board meetings and in the data analysis process. Each CEG will have at least six members and have membership ensuring representation across age, gender, Aboriginal and Torres Strait Islander heritage, ethnicity and locality (ie, regional, remote, rural and metropolitan).

The study and CEG will include people with intellectual disability across the full range of support needs, from those who live relatively independently with minimal support to those who require extensive or pervasive support in most areas of daily living. We use support-needs language rather than clinical severity classifications, consistent with a strengths-based approach that recognises each person’s unique requirements. To support the meaningful participation of people with different support needs in the CEGs, we will provide a range of accommodations. These include Easy Read materials for all written documents, visual supports, extended time for discussions, accessible meeting formats (including online and in-person options), co-facilitation by researchers with intellectual disability and the option for participants to bring a trusted person to meetings. Arts-based methods such as photovoice and body mapping will also be offered as alternative ways to contribute for people who prefer non-verbal or creative modes of communication.

The researchers conducting the interviews will be of mixed backgrounds and experienced in inclusive research. They include people with intellectual disability, researchers in disability and/or education studies, genomics, genomics education, psychosocial researchers and teachers.

### Engagement structure

Our engagement structure includes overlapping groups to ensure rich and varied perspectives (figure 1). The GeneEQUAL Advisory Board is made up of 48 members, including CEG representatives, government and professional organisation members, disability advocacy and support groups, experts in trauma-informed care and an Aboriginal and Torres Strait Islander research group. The Guiding Principles Development Group (GPDG) includes members with intellectual disability, family members, healthcare workers and researchers.

All people with intellectual disability will be remunerated via a gift card for their time and expertise in accordance with Australian guidelines.

#### Patient and public involvement

People with intellectual disability and their families/support people will be included from the beginning of this study. The research questions and project plan are informed by the priorities, experiences and preferences identified in previous work^8 28^ and by GeneEQUAL’s research team. GeneEQUAL involves people with intellectual disability as chief and associate investigators and Advisory Board members, with key roles in co-designing and co-leading the study. GeneEQUAL builds on a decade-long research partnership between IS and JLR, a self-advocacy leader and a researcher with intellectual disability. This collaborative relationship has evolved into authentic co-leadership across 22 funded projects. JLR and IS have also co-mentored other people with intellectual disability into research roles, creating a cascading model where experienced researchers with intellectual disability guide newcomers. This demonstrates that meaningful co-production emerges from sustained relationships, mutual respect and genuinely shared research leadership.

People with intellectual disability will help to choose outcome measures and will be essential in participant recruitment. They will play key roles in developing the dissemination plans, including Easy Read summaries, short videos and accessible newsletters.

### Project design

Our previous work co-designing the GeneEQUAL Educational Toolkit in the state of New South Wales with people with intellectual disability^8^ informed the design of this current study. Our rationale and assumptions in designing this programme were based on this previous successful work.

**Figure 2:**
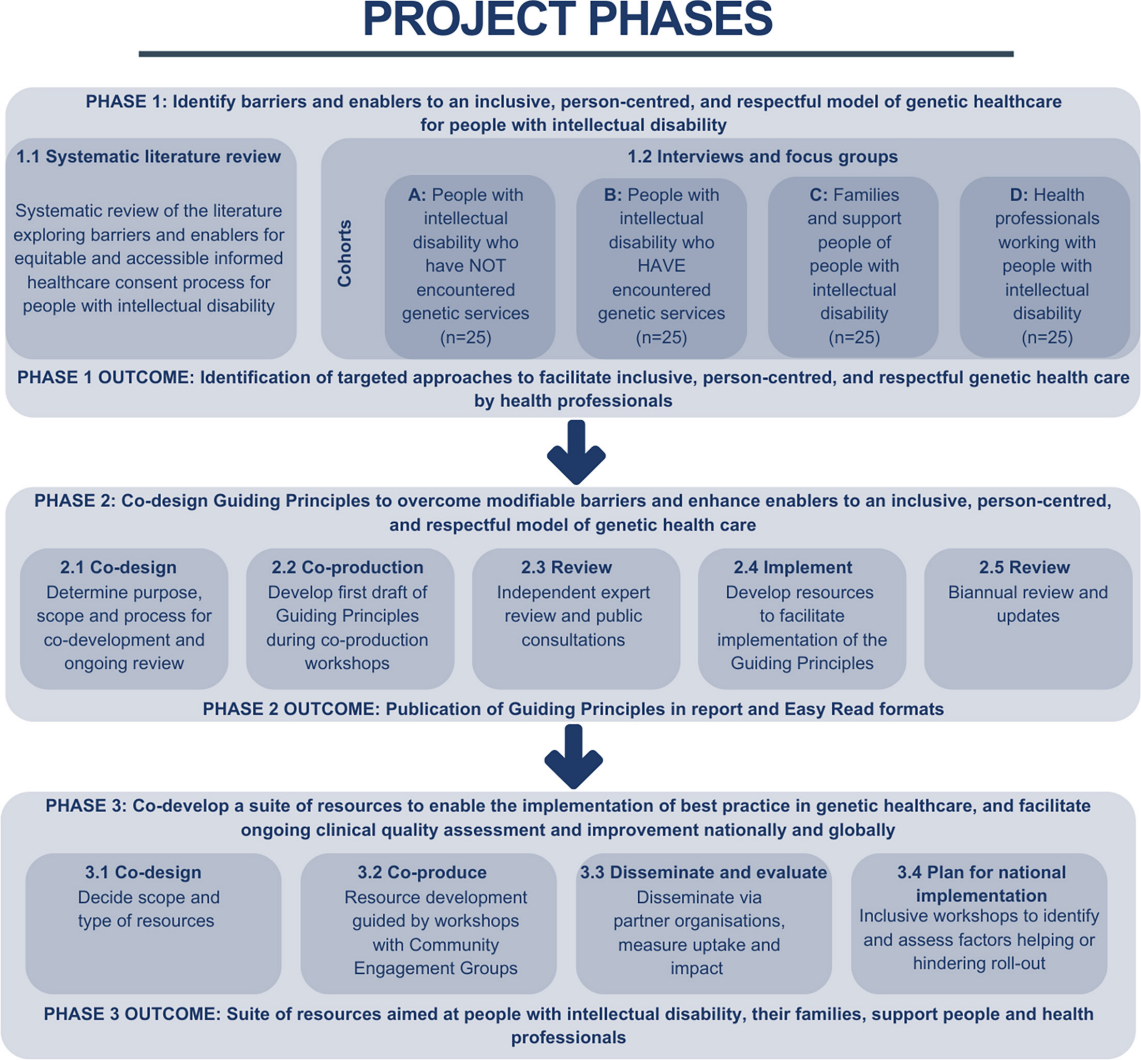
Project phases.

This three-phase qualitative study progresses from understanding to action to implementation (figure 2). Each phase builds on previous findings while maintaining the continuous involvement of people with intellectual disability. The project will run from the time of Phase 1 ethics approval until October 2028, with data collection anticipated to occur shortly after the programme starts until January 2028.

#### Phase 1: exploring barriers and enablers to inclusive genetic healthcare

We will recruit four groups totalling approximately 100 people to participate in qualitative research to help us explore in depth barriers and enablers to inclusive, person-centred and respectful genetic healthcare. The groups will be (1) people with intellectual disability with genetic testing and/or counselling experience; (2) people with intellectual disability without genetic testing and/or counselling experience; (3) family members/support people and (4) healthcare professionals. We will use convenience sampling but will strive to maximise diversity across age, gender, ethnicity and location.

To ensure all participants can contribute, we will offer flexible and accessible ways to participate, including interviews or focus groups (online or in person), in-person arts-based methods (photovoice and body mapping)^38 39^ and yarning circles for Aboriginal and Torres Strait Islander participants. Yarning in groups (ie, a yarning circle) or in one-on-one conversations (ie, ‘a yarn’) is a recognised qualitative research method and offers an alternative for Aboriginal and/or Torres Strait Islander people who prefer these.^40 41^ We estimate that interviews and focus groups will last 30–60 min; they will be audio-recorded (with informed consent) and transcribed. Interview and focus group protocols will be co-designed through an iterative process, drawing on literature review findings, previous GeneEQUAL studies and collaborative input from research team experts, including researchers with intellectual disability, family members/support people and healthcare professionals. This ensures protocols are accessible to people with intellectual disability or other diverse needs, academically grounded and experientially relevant. The full interview and focus group protocols are available from the corresponding author.

We will use inductive content analysis, a qualitative method well-suited to exploratory research.^42^ The analysis will follow a five-step process: (1) familiarisation through thorough reading of all transcripts; (2) open coding to identify units of meaning; (3) category development, where categories and subcategories are developed in parallel through iterative refinement of the codes; (4) comparison and refinement of all categories and subcategories and (5) thematic abstraction to develop overarching themes and subthemes that synthesise the categories that can answer our research questions.^43^

Data from arts-based methods will be captured through multiple channels. For photovoice, participants will take or select photographs representing their experiences and discuss the meaning and significance of each photograph with a researcher. These discussions will be audio-recorded and transcribed alongside the photographs themselves. For body mapping, participants’ visual representations will be documented through high-resolution photography and accompanied by participant narratives explaining their choices, which will also be audio-recorded and transcribed. The visual data (photographs and body maps) will be analysed alongside the transcripts, with attention to both the visual content and participants’ interpretations of their own work.

The yarning circles and individual yarns will be audio-recorded (with informed consent) and transcribed. Yarning will be led by Indigenous researchers and will follow culturally appropriate protocols, including allowing conversations to flow naturally rather than adhering to a rigid interview schedule. All transcribed data from arts-based methods and yarning will be incorporated into the inductive content analysis described above, alongside data from interviews and focus groups, ensuring that all forms of data contribute equally to the findings.

To enhance rigour, multiple researchers will code independently, followed by meetings to reach consensus on data organisation.^44^ Field notes and mind maps will be used to prompt data analysis.^45^ Regular team discussions and participant validation will ensure findings remain grounded in participants’ experiences.

#### Phase 2: co-designing Guiding Principles for inclusive genetic healthcare

The GPDG will be guided by Phase 1 findings. Meetings will be accessible, and materials will be developed in Easy Read to ensure people with intellectual disability can contribute fully.

Development and evaluation will follow the Appraisal Guidelines for Research and Evaluation (AGREE) II-compliant processes,^46^ using the Grading of Recommendations, Assessment, Development and Evaluation (GRADE) framework.^47^ The draft principles will be evaluated by expert review and consultations. When analysing the collected feedback, we will use researcher triangulation (where several team members independently review and code the same data) and member checking (where participants confirm whether our interpretations of the data accurately reflect their perspectives) to ensure consistency and reliability.

To ensure the national Guiding Principles are a ‘living document’, regular biannual meetings of the GPDG will be held throughout the project to update the Guiding Principles, guided by our Advisory Board and CEGs.

#### Phase 3: co-develop a suite of resources for the implementation of best practice

Using our pilot study findings and stakeholder input, we will co-develop a suite of resources, including educational materials such as videos, Easy Read social stories and fact sheets; communication tools for people with high support needs and training resources for genetic healthcare professionals. CEG workshops will guide our decisions about content, media and design preferences. These workshops and associated materials will be in a format that is accessible to people with intellectual disability.

We will evaluate the impact of the resources on people with intellectual disability via focus groups conducted during the co-design process and accessible interviews conducted once the resources are completed. Focus groups will be recorded, transcribed verbatim and analysed using inductive content analysis.

The impact of the resources on families/carers will be evaluated through online surveys and interviews after they have interacted with the resources. The survey incorporates questions from the Patient Activation Measure (PAM) and Genomics Outcome Scale (GOS) items^48 49^ and may be updated during the co-design process in line with identified needs and priorities. These measures were chosen because they will capture the impact of the resources on each individual’s readiness and ability to take an active role in their family member’s health and healthcare (PAM) and the personal and psychosocial impact of the resources (GOS).

Post-pre surveys, similar to those used to assess the toolkit developed as part of our pilot study and based on the Capability Opportunity Motivation and Behaviour framework, will explore whether healthcare professionals’ capability, motivation and opportunity to apply best practices have changed after using the resources.^8^

The resources will be rolled out nationally, facilitated by the Investigators and Advisory Board, with the help of their wide and diverse networks. We will conduct an Intervention Scalability Assessment^50^ with 30–40 stakeholders at inclusive workshops across all Australian states and territories to identify factors that help or hinder the implementation and use of the resources. The implementation strategy will be revised in line with these findings, as needed.

### Sample size and data saturation

Our sample size is based on established principles for qualitative research.^51^ For Phase 1 data collection, we will aim for approximately 100 participants across the four stakeholder groups, with around 25 people per group. Recent systematic reviews show that qualitative research consistently reaches a point defined as saturation within 9–17 interviews or 4–8 focus group discussions, particularly for studies with similar populations and clearly defined objectives.^52 53^ Data saturation, the point at which new data becomes redundant and repeats what was previously expressed, is particularly relevant to inductive content analytic approaches.^42^ Our pilot study with 15 participants with intellectual disability reached data saturation, which supports our proposed sample size.^28^ Applying these principles to data collection, if new data repeat what was expressed in previous data, with no new content categories or themes emerging after three additional interviews, we will stop recruitment for that group. However, if new themes or content categories are identified, we will extend recruitment up to 35 per group to ensure comprehensive coverage.^52^

For Phase 2, each GPDG will have four experts, which will be sufficient to provide a broad perspective and expertise in the development of the Guiding Principles. The Advisory Board has 48 members.

For Phase 3, each CEG involved in developing resources will comprise at least 6 participants (18 in total). This aligns with focus group methodology to attain diverse community feedback while ensuring a comfortable group environment.^54 55^ The multi-stakeholder evaluation of the co-produced resources will recruit a minimum of 25 participants per stakeholder group (matching Phase 1 cohorts) to obtain diverse community feedback, reflecting common qualitative research practice.

## Ethics and dissemination

### Ethics approvals

Ethics approval was granted by the UNSW Sydney Human Research Ethics Committee (HC230353) and the Australian Institute of Aboriginal and Torres Strait Islander Studies (REC-0284). Our approach respects Indigenous data sovereignty principles, with Indigenous leadership guiding how information is collected, interpreted and shared.

### Informed consent

We use a voluntary written informed consent process for all participants, including those with intellectual disability. This approach is based on extensive feedback from people with intellectual disability that assuming incapacity to consent is offensive and risks triggering post-traumatic stress from previous experiences where they were required to prove their capacity.^8^ The project team includes co-researchers with intellectual disability, reflecting our commitment to people with intellectual disability being active participants in research decisions. We will use a continuous consent process and include reasonable adjustments such as extended time and Easy Read and plain English participant information sheets and consent forms, as appropriate. We will use the Teach-back method to ensure understanding, where participants explain information they have been given in their own words, researchers clarify any misunderstandings and this process continues until comprehension is achieved.^56^ Participants will be encouraged to bring someone they trust to interviews and will be assured that they can withdraw at any time without affecting their relationship with researchers.

### Data management

Data will be audio-recorded and transcribed by the research team, with all identifiers removed. Pseudonyms will replace names and the master de-identification list will be stored separately from anonymised data on secure, password-protected systems accessible only to team members.

### Safety monitoring

The research team is experienced in working with people with intellectual disability. We recognise there are potential harms associated with genetic healthcare in people with intellectual disability, such as uncomfortable or worrying resonances with historical eugenics programmes or the possibility that individuals may perceive themselves as ‘faulty’ if a genetic variant is responsible for their intellectual disability.

Although no concerns were identified during our pilot project, to minimise the risk of potential harm, interviews will be conducted within a trauma-informed framework by a co-researcher with intellectual disability and/or a researcher with many years’ experience working with people with intellectual disability and a language guide will be applied across the project to ensure emotive or negative language is not used. A safety protocol is in place to manage any participant distress, including pausing or stopping the interview/focus group, debriefing after the session and arranging appropriate support if needed through family members, support workers or general practitioners.

We will implement measures to support self-care and researcher safety and well-being to build researcher resilience and prevent vicarious trauma.^57^

### Dissemination plan

All materials will be available on our accessible websitee (www.GeneEQUAL.com). Findings will be disseminated through peer-reviewed publications, conference presentations, health professional and self-advocacy websites and social media. Participants will receive accessible summaries of findings via email or video, with researchers available to answer questions as necessary.

## Discussion

The GeneEQUAL project addresses a critical gap in healthcare equity by developing the world’s first inclusive, person-centred genetic healthcare model for people with intellectual disability. Our pilot research revealed that people with intellectual disability frequently feel unsupported in genetic healthcare, with numerous missed opportunities for obtaining genetic diagnoses that could inform practical healthcare plans. This protocol outlines our approach to transforming this situation through rigorous co-production methodologies.

The strength of our approach lies in genuine co-production, where people with intellectual disability contribute as active researchers rather than passive subjects. Our three-phase design deliberately moves from understanding barriers to developing and then implementing solutions. Using different data collection methods, including arts-based approaches^38 58^ and yarning circles,^59^ allows people to participate in line with their communication preferences and ways of being, knowing and doing.^60^ The resulting Guiding Principles will be distinctive in having emerged directly from lived experience rather than from professional assumptions of what people need.

We anticipate several practical outcomes. For people with intellectual disability, the resources will support meaningful participation in healthcare decisions. For their families and support people, it will enable accessible and strengths-based communication about genetics. For healthcare professionals, the project will provide evidence-based approaches to inclusive healthcare. For healthcare systems, examining how to implement a more inclusive model of genetic healthcare will identify pathways for scaling these approaches nationally.

A central challenge is that meaningful co-production is resource-intensive and requires sustained commitment from all stakeholders. Our layered engagement structure aims to address this by distributing responsibility across the CEGs. Additionally, the heterogeneity of intellectual disability means resources must be adaptable to different support needs and cultural contexts, which our emphasis on multimodal resources and iterative testing aims to address.

While focused on Australia, this work has broad international relevance. The exclusion of people with intellectual disability from advances in genetic healthcare is a global phenomenon. Our methodological approach offers a template that could be adapted to other healthcare systems and may also inform co-production approaches in other clinical fields where people with intellectual disability face similar barriers to healthcare participation.

In the long term, this research will foster a paradigm shift from health research that is done *on* people with intellectual disability to that which *is led by* people with intellectual disability. It can also serve as a model for other areas of health, given the urgent need for radical improvement in all healthcare for people with intellectual disability. The mentoring of co-researchers through our programme will also grow the next generation of researchers with intellectual disability.

10.1136/bmjopen-2025-110086.supp1 Supplementary data

## Supplementary Material

Reviewer comments

Author's manuscript
