# Supplementary material for: Building genetic healthcare together: an Australian co-production three-phase mixed-methods research protocol with people with intellectual disability
Source: BMJ Open. 2026 Jul 10;16(7):e110086. doi: 10.1136/bmjopen-2025-110086 (PMC13358344; doi:10.1136/bmjopen-2025-110086)
Supplement: Supplementary data [file bmjopen-16-7-s002.pdf]

SUPPLEMENTAL MATERIAL

Easy Read Summary

Making Genetic Health Care Better For People With Intellectual Disability

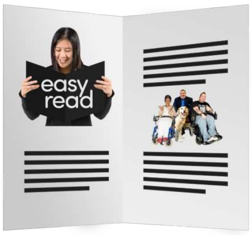

This booklet is to explain a **research study**

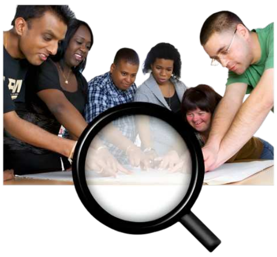

A **research study** is something we do

- when we want to find information

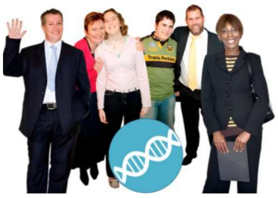

This research study will be about

- people with intellectual disability and
- genetic health care

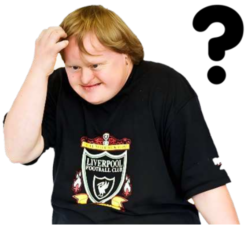

Some things in the booklet can be hard to understand

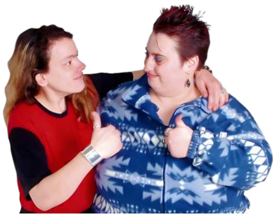

You can read it with someone you trust

For example, a family member, friend, or

Strnadová I, *et al. BMJ Open* 2026; 16:e110086. doi: 10.1136/bmjopen-2025-110086

health care worker

What is genetic health care?

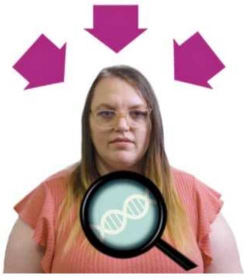

Genetic health care is for people who

- have a **genetic condition**
- might have a genetic condition
- want to know more about genetic conditions and tests

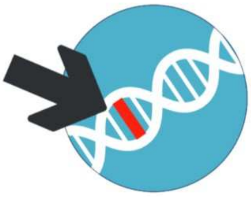

A **genetic condition** happens if a gene changes

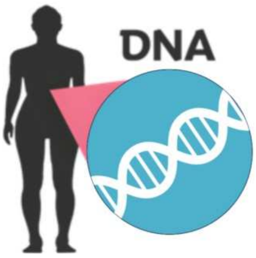

Genes are the things our body

- that make us the way we are

Genetic health care is important for all people

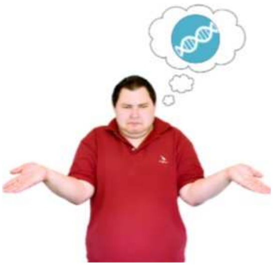

This is because we may have a genetic condition

- and we do not know about it

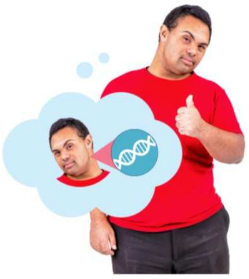

If we know about our genetic condition we can

- understand things about us
- have better health treatment
- prepare for future problems

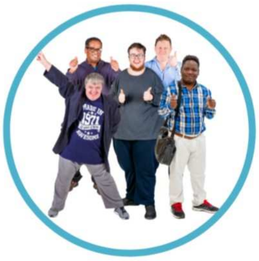

It can also help us to

- meet people with same genetic condition
- make decisions
- have a good life

Why are we doing this study?

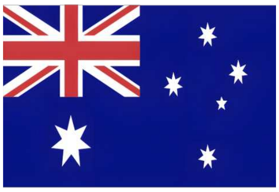

In 2007 Australia signed a document called

- United Nations Convention on the Rights of People with Disabilities

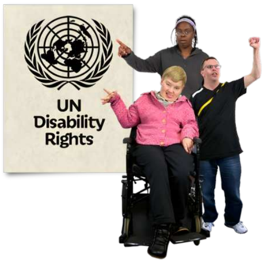

Australia wants people with disability

- to have the same rights as everyone else

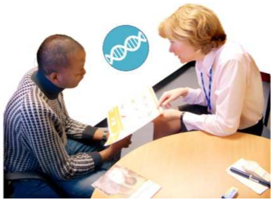

Having the same rights includes

- having the best possible genetic health care

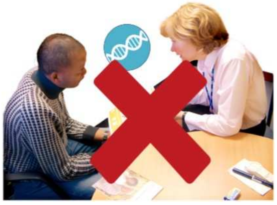

But people with intellectual disability do not have

- the best genetic health care

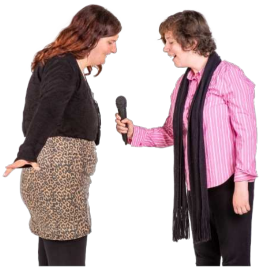

In our previous study, we asked people with intellectual disability

- about their experience with genetic health care

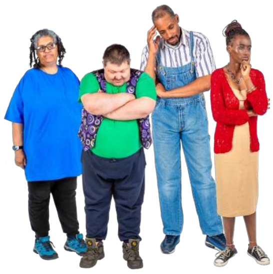

They told us they want support

They do not feel included and feel sad

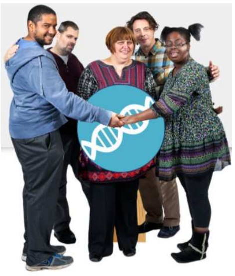

People want to

- understand genetic health care
- be treated with respect
- have the right resources

4

What do we want to do in this study?

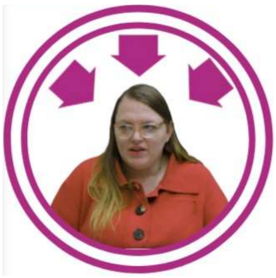

We want to make genetic health care

- **inclusive**
- **person centred**
- **respectful**

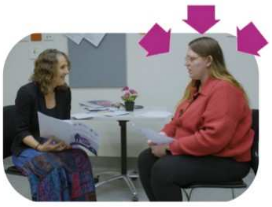

Inclusive, person centred and respectful

means that health care workers

- listen to you
- treat you well
- give you time to understand
- help you make the right decision

How are we going to do it?

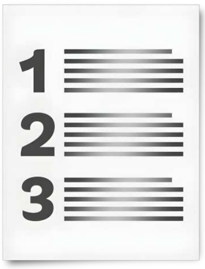

We will do 3 things

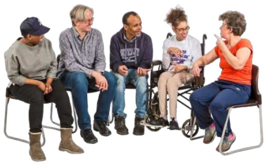

We will ask people about genetic health care

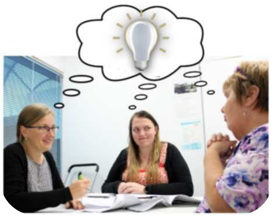

Together we will think about answers

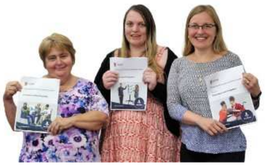

We will share our answers

We will make resources to support health care workers

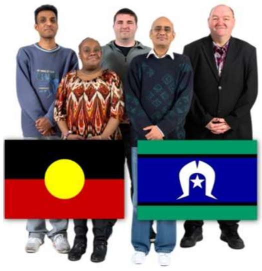

We will work with people with intellectual disability

- who are Aboriginal and Torres Strait Islander

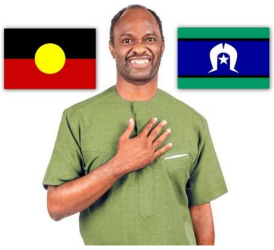

We will make sure to be **culturally safe**

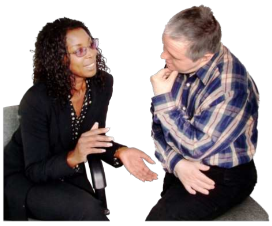

Culturally safe means we

- respect the history of people
- listen and ask about their preferences
- help people feel comfortable

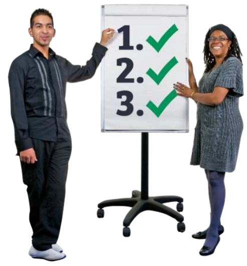

Our study will happen in 3 steps

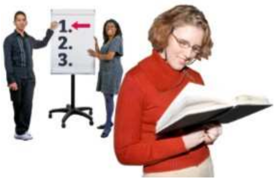

Step 1

We will find what other people did

- to improve genetic health care

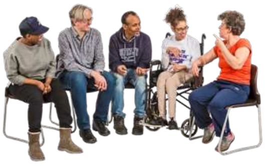

We will talk to 25 Australians from each group

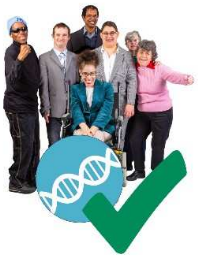

Group 1 will be people with intellectual disability

- who had experience with genetic health care

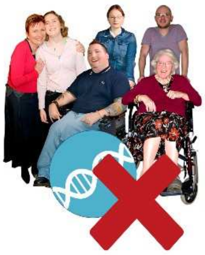

Group 2 will be people with intellectual disability

- who did not have experience with genetic health care

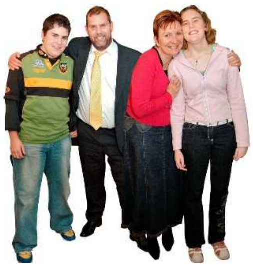

Group 3 will be family members and carers

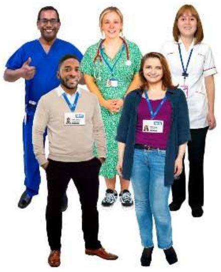

Group 4 will be health care workers

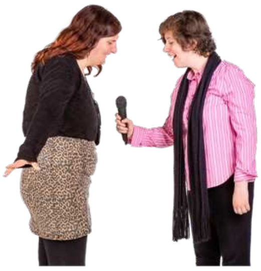

We will use interviews, focus groups and **yarning circles**

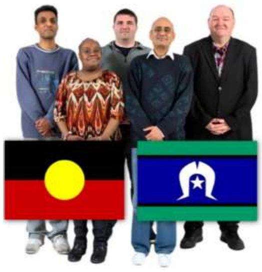

Yarning circles are something that Aboriginal and Torres Strait Islander do to

- share stories
- talk to each other

Strnadová I, *et al. BMJ Open* 2026; 16:e110086. doi: 10.1136/bmjopen-2025-110086

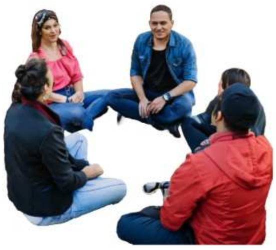

In yarning circles

- people sit in a circle
- everyone gets a turn to speak

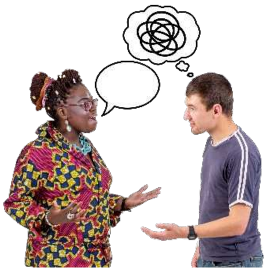

We will also use other things

- to support people to share their ideas with us

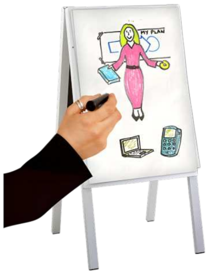

People can make body maps to tell us what they think

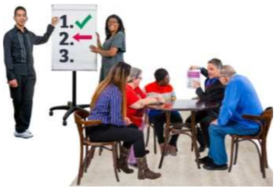

Step 2

We will

- talk about what people told us
- think about answers and ideas

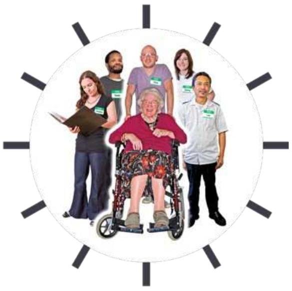

Representatives of

- people with intellectual disability
- their families and carers
- health care workers

will be part of Step 2

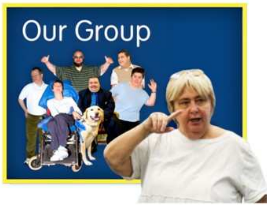

Representatives are people

- who speak for their group
- share what their group thinks
- make sure their needs are heard

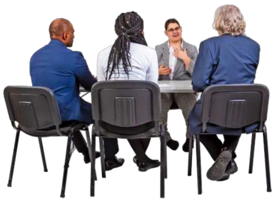

We will also ask experts in intellectual disability

- what they think about our answers and ideas

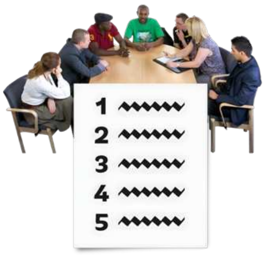

We will then write **Guiding Principles** to support health care workers

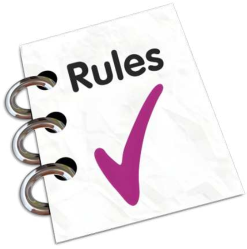

Guiding Principles are similar to rules

Strnadová I, et al. BMJ Open 2026; 16:e110086. doi: 10.1136/bmjopen-2025-110086

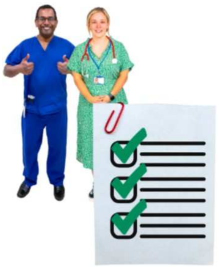

They will tell health care workers

- what is the right thing to do

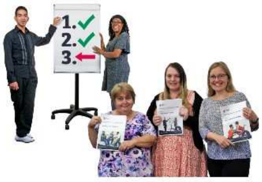

Step 3

We will make and share resources

- to help make genetic health care better

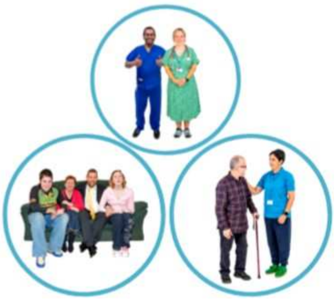

These resources will be for

- people with intellectual disability
- their families and carers
- health care workers

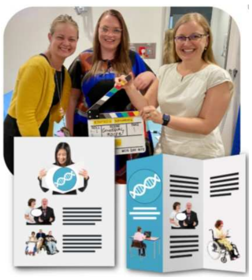

Examples of resources are

- videos
- Easy Read materials
- social stories

11

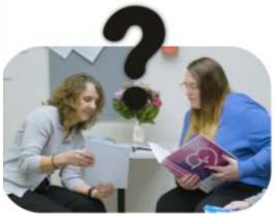

We will also check if

- people are using the resources
- the resources help them

Doing things the right way

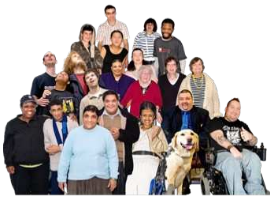

A lot of people will be involved in this research study

There will be

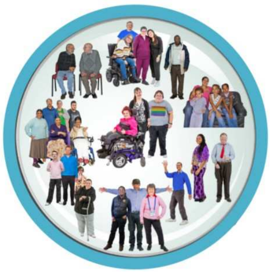

- participants
- co-researchers
- representatives
- researchers
- families
- health care workers

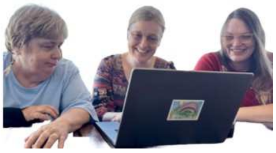

Some people with intellectual disability are co-researchers in our study

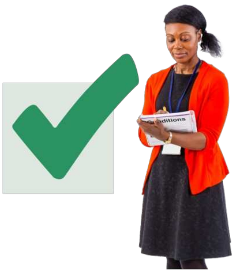

Everyone in our study needs to be safe

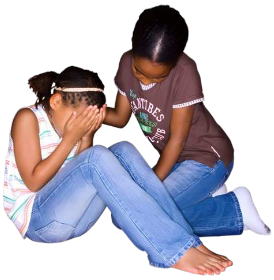

Some people with intellectual disability

- had very bad experiences in their life

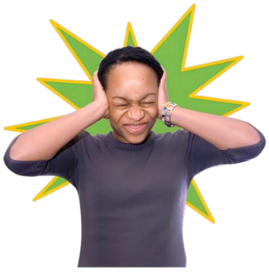

Talking about genetic health care can make them upset

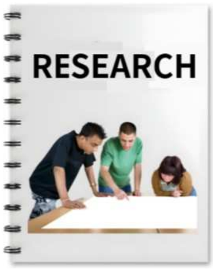

That is why in this study we use a **trauma informed approach**

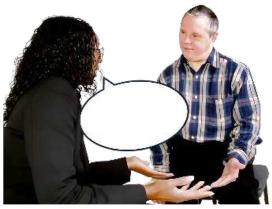

Trauma informed approach is about people feeling safe and respected

Strnadová I, *et al. BMJ Open* 2026; 16:e110086. doi: 10.1136/bmjopen-2025-110086

We support people if they need to

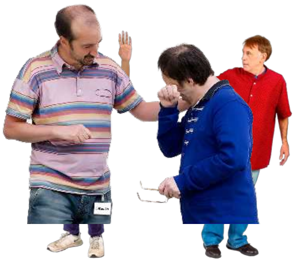

People can also leave our study if they want

Who is doing this research study?

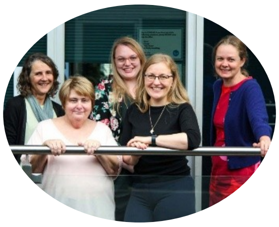

The **GeneEQUAL team** is doing this study

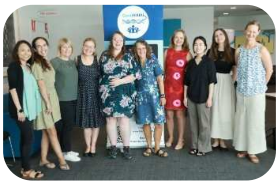

The team includes people

- with intellectual disability
- with other disability
- academic researchers
- health care workers
- teachers

Strnadová I, *et al. BMJ Open* 2026; 16:e110086. doi: 10.1136/bmjopen-2025-110086

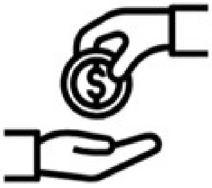

We got funding from the Australian Federal Government (NHMRC)

Where will I see the results?

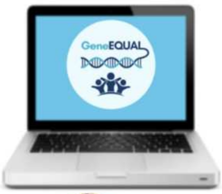

Everything will be available on our website

www.geneequal.com

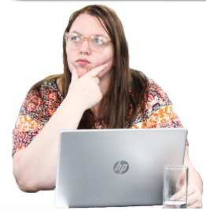

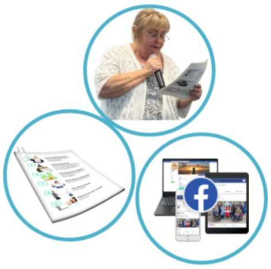

We will also

- write articles
- go to conferences
- publish on other websites
- share in social media

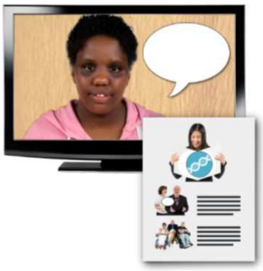

Participants will get our findings in Easy Read and videos

About this booklet

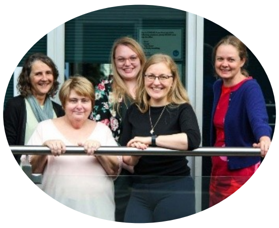

The GeneEQUAL team made this booklet

It was made using Photosymbols and Adobe

Professional Licence 708948325

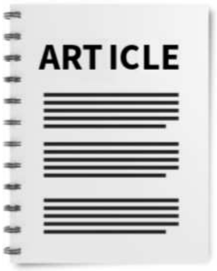

The full article is called:

Developing inclusive genetic healthcare for people with intellectual disability: A co-production research protocol

Strnadová I, *et al. BMJ Open* 2026; 16:e110086. doi: 10.1136/bmjopen-2025-110086
