# Supplementary figures and images for: Building genetic healthcare together: an Australian co-production three-phase mixed-methods research protocol with people with intellectual disability
Source: BMJ Open. 2026 Jul 10;16(7):e110086. doi: 10.1136/bmjopen-2025-110086 (PMC13358344; doi:10.1136/bmjopen-2025-110086)

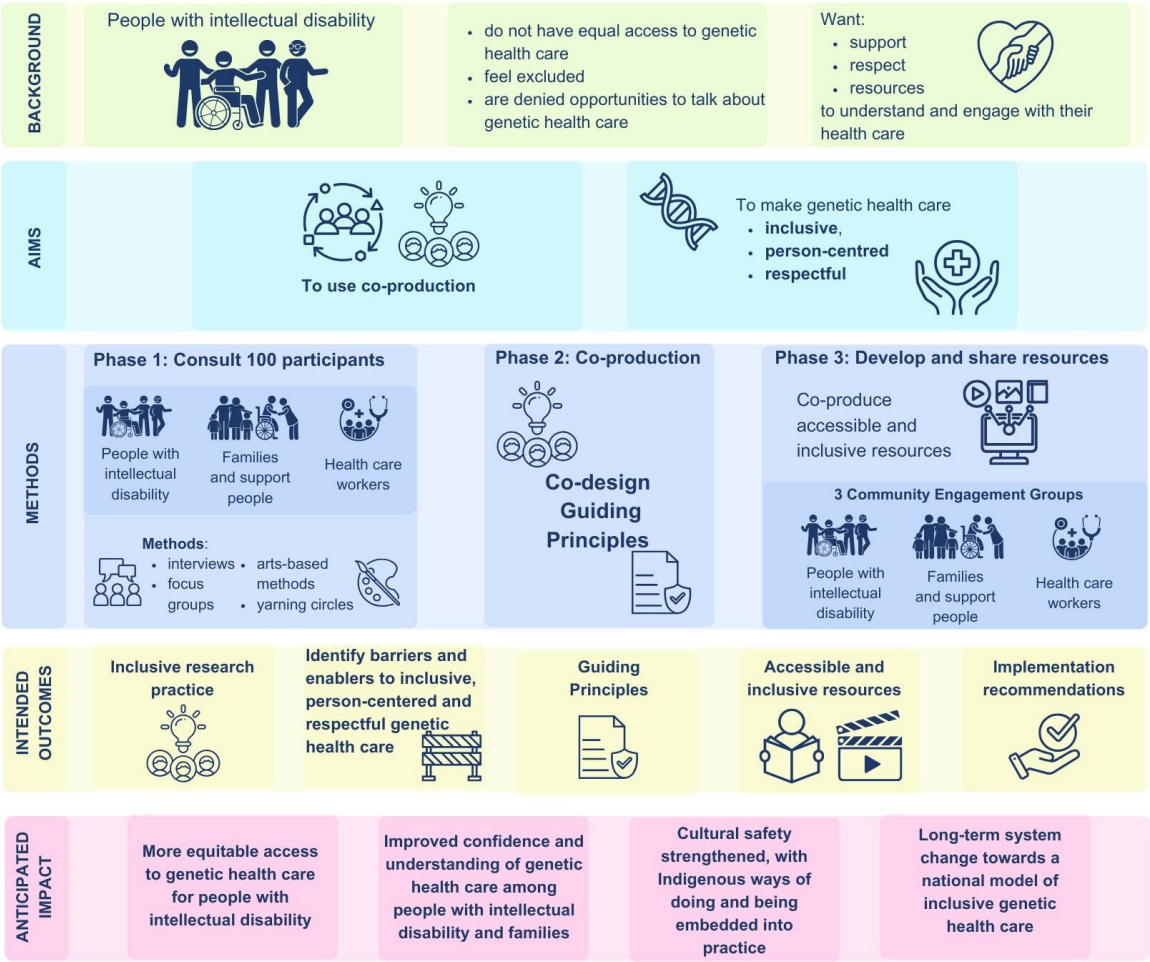

Supplement: Supplementary data [file bmjopen-16-7-s001.pdf]
