# Supplementary material for: Building genetic healthcare together: an Australian co-production three-phase mixed-methods research protocol with people with intellectual disability
Source: BMJ Open. 2026 Jul 10;16(7):e110086. doi: 10.1136/bmjopen-2025-110086 (PMC13358344; doi:10.1136/bmjopen-2025-110086)
Supplement: Reviewer comments [file bmjopen-2025-110086.reviewer_comments.pdf]

PEER REVIEW HISTORY

BMJ Open publishes all reviews undertaken for accepted manuscripts. Reviewers are asked to complete a checklist review form and are provided with free text boxes to elaborate on their assessment. These free text comments are reproduced below.

ARTICLE DETAILS

Title (Provisional)

Building genetic healthcare together: An Australian co-production three phase mixed-methods research protocol with people with intellectual disability

Authors

Strnadová, Iva; Tso, Michelle; Loblinzk Refalo, Julie; Roberts, Natalie; Danker, Joanne; Sarfaraz, Skie; Boyle, Jackie; Terrill, Bronwyn; Halliburton, Celia; Pantoja Mardones, Claudia; Hayes, Sarah; Hurd, Sam; King, Caitlin; Barlow-Stewart, Kristine; Evans, Elizabeth; Leonard, Helen; Mar Fan, Helen; Rodgers, Jonathan; Vella, Yvette; Mcgaughran, Julie; Best, Stephanie; Turbitt, Erin; Leach Scully, Jackie; Pratt, Greg; Palmer, Elizabeth E

VERSION 1 - REVIEW

**Reviewer**	**1**
**Name**	**Kelly, Dervla**
**Affiliation**	**University of Galway College of Medicine Nursing and Health Sciences, School of Pharmacy and Medical Sciences**
**Date**	**06-Dec-2025**
**COI**	**None**

Thank you for the opportunity to review the protocol for 'Developing inclusive genetic healthcare for people with intellectual disability'. Overall, it is a very well written protocol describing a proposed development of a national model of genetic healthcare. The following comments are items for clarification.

As per BMJ Open requirements, please add dates of proposed study.

Page 10 Description of Three Community Engagement Groups (CEGs): Please add proposed number participants from each of 3 groups?

Please describe the range of ID to be included – will it include people with mild , moderate, severe intellectual disability? Could you please briefly describe some accommodations to enable participation of people with different levels of intellectual disbaility in the community engagement groups. Will the groups be mixed or not? – I believe not based on the diagram. It would be useful to understand the rationale for this.

Page 10 Number of people on the GeneEQUAL advisory board?

Page 10, 11 PPI section – well written and a nice description of genuine partnership

Page 14: Phase 3 provides a description of the development of resources and proposes to evaluate the impact of the resources with healthcare professionals. There is some ambiguity on whether the measurement will focus on evaluating the guiding principles/ model of genetic services, the resources (reach etc…) or something else?

Page 14:

Could reference for adapted Patient Activation Measure be checked (no. 49).

Could a rationale be added for the selection of each the survey instrucments / questions.

Did the authors consider measuring the impact of resources on the target audience (people with intellectual disability / carers)?

Page 14 In order to assess scalability, it is useful to have a plan for the immediate roll out of the resources and guiding principles. How do the authors envisage the model of care will be rolled out? Nationally or in a pilot region/centre? Is the 'intervention' the guiding principles or the resources developed or something else?

Section 3.4 sample size: unclear if this section refers to surveys / interview for Patient Activation Measure, Genomics Outcome Scale, HCP motivations or scalability assessment? Please clarify sampling and data collection approaches for each instrument.

Will the instruments need to be adapted for people with intellectual disability or other accommodations made during data collection?

Reviewer 2

Name Tilley, Elizabeth

Affiliation The Open University, School of Health, Wellbeing and Social Care

Date 16-Dec-2025

COI None

This is a very comprehensive and robust protocol, setting out a clear road map for the delivery of an ambitious co-produced research project that aims to address a significant gap in the provision of equitable healthcare for people with intellectual disability. Although the research is Australia specific, the protocol is likely to be of significant interest to researchers in other countries given its focus on such a clinically, socially and ethically complex area of healthcare. The protocol sets out a model of best practice in terms of its theoretical, methodological and ethical approach to co-production and inclusive healthcare research for people with intellectual disabilities.

There are two main areas that I would appreciate further detail on prior to publication:

1) The authors refer to the inclusion of people with 'high support needs' in the research. Can this term please be defined/explained as its understanding and scope can vary significantly within and across national contexts. For example, is the intention to include people with profound and multiple intellectual disabilities in the research (and /or the perspectives of those who support them)?

2) The protocol explains the potential benefits of genetic testing and genetic counselling for people with intellectual disabilities, identifying the health and wellbeing impacts when this is denied. I wondered if the authors might also reflect on any potential harms that might be associated with genetic healthcare for this population. For example, genetic screening and testing for some people with intellectual disabilities and their families may have uncomfortable/worrying resonances with historical eugenics programmes. Did any such concerns arise during the pilot project? If so, how were they addressed (and how did this feed into the current research)? Does the team anticipate such questions arising in the main research and if so, what mechanisms would be in place to support participants through such discussions? This could perhaps be captured as part of Section 4, Ethics and Dissemination.

In addition, I have identified a few other points of clarification for the authors to address:

a. Abstract: Under Methods and analysis, please add a few words to explain the Purpose of Phase 1, ie to identify barriers and enablers. In Phase 3, please clarify what is meant by 'investigate national roll-out'. I think you are referring here to the factors affecting implementation, but it would be good to make this clear.

b. Introduction: In the first paragraph when you write about misattributing health problems to disability, I wonder if it might be helpful to explicitly include the term 'diagnostic overshadowing'. Given this project is specifically addressing the genetic/biological component of some intellectual disabilities, it's particularly important to draw attention to those instances in which health issues/conditions are missed because of inappropriate/misplaced assumptions about the association between disability and symptoms/behaviour(s).

c. Introduction: Third paragraph, as above, please define 'high support needs'.

d. Methods and Analysis: p.12, section 3.3.1. Please explain how you will capture and analyse data relating to arts-based methods and Yarning.

e. Methods and Analysis: p.14, 3.3.3: Please clarify what will you do with the data collected about what helps/hinders implementation. For example, is it your intention to revise the implementation strategies as a result of your findings?

VERSION 1 - AUTHOR RESPONSE

Reviewer 1

Thank you for the opportunity to review the protocol for 'Developing inclusive genetic healthcare for people with intellectual disability'. Overall, it is a very well written protocol describing a proposed development of a national model of genetic healthcare. The following comments are items for clarification.

Response: Thank you for your kind words and your invaluable feedback.

1. As per BMJ Open requirements, please add dates of proposed study.

Response:

As noted above, the study commences when ethics approval is obtained for the Phase 1 and will end in October 2028. This has been added to the manuscript as shown below:

> "The project will run from the time of Phase 1 ethics approval until October 2028, with data collection anticipated to occur shortly after the program starts until January 2028."

2. Page 10 Description of Three Community Engagement Groups (CEGs): Please add proposed number participants from each of 3 groups?

Response:

We have updated the manuscript as shown below:

> "Each CEG will have at least 6 members and have membership ensuring representation across age, gender, Aboriginal and Torres Strait Islander heritage, ethnicity, and locality (i.e. regional, remote, rural and metropolitan)."

3. Please describe the range of ID to be included – will it include people with mild , moderate, severe intellectual disability? Could you please briefly describe some accommodations to enable participation of people with different levels of intellectual disbaility in the community engagement groups. Will the groups be mixed or not? – I believe not based on the diagram. It would be useful to understand the rationale for this.

Response:

Thank you for this important clarification. We have updated Section 3.1 to address all three points:

Range of intellectual disability:

"The study and Community Engagement Group will include people with intellectual disability across the full range of support needs, from those who live relatively independently with minimal support to those who require extensive or pervasive support in most areas of daily living. We use support-needs language rather than clinical severity classifications, consistent with a strengths-based approach that recognises each person's unique requirements."

Accommodations:

"To support the meaningful participation of people with different support needs in the CEGs, we will provide a range of accommodations. These include Easy Read materials for all written documents, visual supports, extended time for discussions, accessible meeting formats (including online and in-person options), co-facilitation by researchers with intellectual disability, and the option for participants to bring a trusted person to meetings. Arts-based methods such as photo-voice and body-mapping will also be offered as alternative ways to contribute for people who prefer non-verbal or creative modes of communication."

Group composition:

"As shown in Figure 1, the three Community Engagement Groups (CEGs) are organised by stakeholder type: (1) people with intellectual disability, (2) families and support people, and (3) healthcare professionals. This structure was chosen to ensure that each group can discuss issues relevant to their specific perspectives and experiences without the potential power imbalances that can arise in mixed stakeholder groups. In particular, separating the group of people with intellectual disability from healthcare professionals and family members ensures that their voices are not overshadowed, and that they can speak freely about their experiences. All three CEGs contribute to the same research phases, and their perspectives are brought together during Advisory Board meetings and in the data analysis process."

4. Page 10 Number of people on the GeneEQUAL advisory board?

Response: We have updated the text to:

> "The GeneEQUAL Advisory Board is made up of 48 members, including CEG representatives, government and professional organisation members, disability advocacy and support groups, experts in trauma-informed care, and an Aboriginal and Torres Strait Islander Research group."

5. Page 10, 11 PPI section – well written and a nice description of genuine partnership

Response:

Thank you, we appreciate your kind feedback

6. Page 14: Phase 3 provides a description of the development of resources and proposes to evaluate the impact of the resources with healthcare professionals. There is some ambiguity on whether the measurement will focus on evaluating the guiding principles/ model of genetic services, the resources (reach etc…) or something else?

Response:

Thank you for pointing this ambiguity out. We have clarified as below:

> "Post-pre surveys similar to those used to assess the toolkit developed as part of our pilot study and based on the Capability Opportunity Motivation and Behaviour (COM-B) framework, will explore whether healthcare professionals' capability, motivation and opportunity to apply best practice have changed after using the resources.[8]"

7. Page 14: Could reference for adapted Patient Activation Measure be checked (no. 49).

Response:

Thank you for noticing this incorrect reference. We have now updated this to:

“49. Hibbard JH, Stockard J, Mahoney ER, Tusler M. Development of the Patient Activation Measure (PAM): Conceptualizing and Measuring Activation in Patients and Consumers. *Health Services Research* 2004;39:1005-26.”

8. Could a rationale be added for the selection of each the survey instrucments / questions.

Response:

Thank you for this suggestion. Additional text has been added to Section 3.3.3:

” The impact of the resources on families/carers will be evaluated through online surveys and interviews after they have interacted with the resources. The survey incorporates questions from the Patient Activation Measure (PAM) and Genomics Outcome Scale (GOS) items,[49,50] and may be updated during the co-design process in line with identified needs and priorities. These measures were chosen because they will capture the impact of the resources on each individual’s readiness and ability to take an active role in their family member’s health and healthcare (PAM), and the personal and psychosocial impact of the resources (GOS).

Post-pre surveys similar to those used to assess the toolkit developed as part of our pilot study and based on the Capability Opportunity Motivation and Behaviour (COM-B) framework will explore whether healthcare professionals’ capability, motivation and opportunity to apply best practice have changed after using the resources.[8]”

9. Did the authors consider measuring the impact of resources on the target audience (people with intellectual disability / carers)?

Response:

Yes, the impact of the resources on people with intellectual disability will be assessed via interviews and focus groups, as surveys are generally not accessible to them.

We have updated Section 3.3.3 to provide more detail about how the impact will be assessed:

"We will evaluate the impact of the resources on people with intellectual disability via focus groups conducted during the co-design process and accessible interviews conducted once the resources are completed. Focus groups will be recorded, transcribed verbatim, and analysed using inductive content analysis."

10. Page 14 In order to assess scalability, it is useful to have a plan for the immediate roll out of the resources and guiding principles. How do the authors envisage the model of care will be rolled out? Nationally or in a pilot region/centre? Is the 'intervention' the guiding principles or the resources developed or something else?

Response:

The resources will be rolled out nationally, facilitated by the members of the Advisory Group and Investigators. The intervention in this context refers to the resources themselves.

We have updated the end of Section 3.3.3:

"The resources will be rolled out nationally, facilitated by the Investigators and Advisory Board, with the help of their wide and diverse networks. We will conduct an Intervention Scalability Assessment[51] with 30-40 stakeholders at inclusive workshops across all Australian States and Territories to identify factors that help or hinder implementation and use of the resources. The implementation strategy will be revised in line with these findings, as needed."

11. Section 3.4 sample size: unclear if this section refers to surveys / interview for Patient Activation Measure, Genomics Outcome Scale, HCP motivations or scalability assessment? Please clarify sampling and data collection approaches for each instrument.

Response:

Thank you for flagging that more information is needed for clarity. We have updated Section 3.4 as shown below:

“Our sample size is based on established principles for qualitative research.[52] For Phase 1 data collection, we will aim for approximately 100 participants across the four stakeholder groups, with around 25 people per group. Recent systematic reviews show that qualitative research consistently reaches a point defined as saturation within 9-17 interviews or 4-8 focus group discussions, particularly for studies with similar populations and clearly defined objectives.[53,54] Data saturation, the point when new data become redundant and repeat what was previously expressed, is particularly relevant to inductive content analytic approaches.[43] Our pilot study with 15 participants with intellectual disability reached data saturation, which supports our proposed sample size.[34] Applying these principles to data collection: if new data repeat what was expressed in previous data, with no new content categories or themes emerging after 3 additional interviews, we will stop recruitment for that group. However, if new themes or content categories are identified, we will extend recruitment up to 35 per group to ensure comprehensive coverage.[53]

For Phase 2, each GPDG will have 4 experts, which will be sufficient to provide a broad perspective and expertise in the development of the Guiding Principles. The Advisory Board has 48 members.

For Phase 3, each CEG involved in developing resources will comprise at least 6 participants (18 in total). This aligns with focus group methodology to attain diverse community feedback while ensuring a comfortable group environment.[55,56] The multi-stakeholder evaluation of the co-produced resources will recruit a minimum of 25 participants per stakeholder group (matching Phase 1 cohorts) to obtain diverse community feedback, reflecting common qualitative research practice.”

12. Will the instruments need to be adapted for people with intellectual disability or other accommodations made during data collection?

Response:

People with intellectual disability will not complete any surveys, therefore they will not need to be adapted. All data collection from people with intellectual disability will be via face-to-face or online meetings. These meetings will be conducted in an accessible way to ensure full and active participation from people with intellectual disability. We have added/updated the text in the following sections to clarify:

Section 3.3.1 paragraph 2:

"To ensure all participants can contribute, we will offer flexible and accessible ways to participate, including interviews or focus groups (online or in person), in person arts-based methods (Photo-voice, body-mapping)[39,40], and yarning circles for Aboriginal and Torres Strait Islander participants. Yarning in groups (i.e. a yarning circle) or in one-on-one conversations (i.e. "a yarn") are a recognised qualitative research method and offer an alternative for Aboriginal and/or Torres Strait Islander people who prefer these.[41,42] We estimate that interviews and focus groups will last 30-60 minutes; they will be audio-recorded (with informed consent) and transcribed. Interview and focus group protocols will be co-designed through an iterative process, drawing on literature review findings, previous GeneEQUAL studies, and collaborative input from research team experts including researchers with intellectual disability, family members/support people, and healthcare professionals. This ensures protocols are accessible to people with intellectual disability or other diverse needs, academically grounded and experientially relevant. The full interview and focus group protocols are available from the corresponding author."

Section 3.3.2 paragraph 1:

"The Guiding Principles Development Group (GPDG) will be guided by Phase 1 findings. Meetings will be accessible, and materials will be developed in Easy Read to ensure people with intellectual disability can contribute fully."

Section 3.3.3 paragraph 1:

"Using our pilot study findings and stakeholder input, we will co-develop a suite of resources including educational materials such as videos, Easy Read social stories, and fact sheets; communication tools for people with high support needs; and training resources for genetic healthcare professionals. CEG workshops will guide our decisions about content, media, and design preferences. These workshops and associated materials will be in a format that is accessible to people with intellectual disability."

Reviewer 2

This is a very comprehensive and robust protocol, setting out a clear road map for the delivery of an ambitious co-produced research project that aims to address a significant gap in the provision of equitable healthcare for people with intellectual disability. Although the research is Australia specific, the protocol is likely to be of significant interest to researchers in other countries given its focus on such a clinically, socially and ethically complex area of healthcare. The protocol sets out a model of best practice in terms of its theoretical, methodological and ethical approach to co-production and inclusive healthcare research for people with intellectual disabilities.

There are two main areas that I would appreciate further detail on prior to publication:

Response: Thank you for your kind words and thoughtful suggestions.

1. The authors refer to the inclusion of people with 'high support needs' in the research. Can this term please be defined/explained as its understanding and scope can vary significantly within and across national contexts. For example, is the intention to include people with profound and multiple intellectual disabilities in the research (and /or the perspectives of those who support them)?

Response:

Thank you for this important point. We have added a definition of 'high support needs' to the manuscript. In the Introduction (paragraph 3), we now state:

> "People with intellectual disability, especially those with high support needs – that is, people who require extensive or pervasive support across most areas of daily living, which includes many people with severe or profound intellectual disability and/or multiple disabilities – experience immense health inequalities, including premature mortality and disproportionate rates of potentially avoidable deaths as a result of late diagnosis or mismanagement.[9-12]"

Our intention is to include people across the full range of support needs. We deliberately use support-needs language rather than clinical severity classifications (e.g., mild/moderate/severe), as this is consistent with a strengths-based, social model of disability that recognises each person's unique support requirements rather than categorising people by perceived deficit. Where direct participation is not possible, we will seek the perspectives of family members and support people who know the person well, as noted in the manuscript. We have added the following to Section 3.1 to explain this distinction:

> "The study will include people with intellectual disability across the full range of support needs, from those who live relatively independently with minimal support to those who require extensive or pervasive support in most areas of daily living. We use support-needs language rather than clinical severity classifications, consistent with a strengths-based approach that recognises each person's unique requirements. "

2. The protocol explains the potential benefits of genetic testing and genetic counselling for people with intellectual disabilities, identifying the health and wellbeing impacts when this is denied. I wondered if the authors might also reflect on any potential harms that might be associated with genetic healthcare for this population. For example, genetic screening and testing for some people with intellectual disabilities and their families may have uncomfortable/worrying resonances with historical eugenics programmes. Did any such concerns arise during the pilot project? If so, how were they addressed (and how did this feed into the current research)? Does the team anticipate such questions arising in the main research and if so, what mechanisms would be in place to support participants through such discussions? This could perhaps be captured as part of Section 4, Ethics and Dissemination.

Response:

Thank you for highlighting this very valid concern.

No concerns arose during the pilot project, and we do not anticipate any arising during this follow-on project. We have added new text into Section 4.4 addressing your excellent points:

> "The research team is experienced in working with people with intellectual disability. We recognise there are potential harms associated with genetic healthcare in people with intellectual disability, such as uncomfortable or worrying resonances with historical eugenics programs, or the possibility that individuals may perceive themselves as 'faulty' if a genetic variant is responsible for their intellectual disability.
>
> Although no concerns were identified during our pilot project, to minimise the risk of potential harm, interviews will be conducted within a trauma-informed framework by a co-researcher with intellectual disability and/or a researcher with many years' experience working with people with intellectual disability, and a language guide will be applied across the project to ensure emotive or negative language is not used. A safety protocol is in place to manage any participant distress, including pausing or stopping the interview/focus group, debriefing after the session, and arranging appropriate support if needed through family members, support workers, or general practitioners."

In addition, I have identified a few other points of clarification for the authors to address:

a. Abstract: Under Methods and analysis, please add a few words to explain the Purpose of Phase 1, ie to identify barriers and enablers. In Phase 3, please clarify what is meant by 'investigate national roll-out'. I think you are referring here to the factors affecting implementation, but it would be good to make this clear.

Response:

Thank you for noting these issues that require clarification. We have updated the abstract as follows:

- Phase 1 description: "Phase 1 will identify barriers and enablers to genetic healthcare for people with intellectual disability by engaging with approximately 100 participants - people with intellectual disability (with and without genetic testing experience), families/support people, and genetic healthcare professionals - through interviews, focus groups, arts-based methods, and yarning circles."

- Phase 3 description: “Phase 3 will develop practical resources using workshops across Australia, guided by three Community Engagement Groups including people with intellectual disability, families/support people, and healthcare providers. This phase will also investigate strategies to optimise national roll-out and implementation of resources among health professionals, people with intellectual disability and their families/carers.”

We have also added further description of Phase 2, which was very short:

> “Phase 2 will co-design and evaluate Guiding Principles, providing solutions to identified barriers and leveraging identified facilitators.”

b. Introduction: In the first paragraph when you write about misattributing health problems to disability, I wonder if it might be helpful to explicitly include the term ‘diagnostic overshadowing’. Given this project is specifically addressing the genetic/biological component of some intellectual disabilities, it’s particularly important to draw attention to those instances in which health issues/conditions are missed because of inappropriate/misplaced assumptions about the association between disability and symptoms/behaviour(s).

Response:

Thank you for making this very important point. We have added the terms ‘diagnostic overshadowing’ into the manuscript, as follows:

> “There is compelling evidence that many health professionals exclude people with intellectual disability from consultations, misattribute health problems to their disability (known as diagnostic overshadowing), and sometimes display unprofessional behaviour.[3,4]”

c. Introduction: Third paragraph, as above, please define ‘high support needs’.

Response:

As noted in our response to Question 1 above, we have added a definition of 'high support needs' in the third paragraph of the Introduction. The same definition is used consistently throughout the manuscript.

d. Methods and Analysis: p.12, section 3.3.1. Please explain how you will capture and analyse data relating to arts-based methods and Yarning.

Response:

Thank you for highlighting this. We have added the following text to Section 3.3.1:

> "Data from arts-based methods will be captured through multiple channels. For photo-voice, participants will take or select photographs representing their experiences, and discuss the meaning and significance of each photograph with a researcher. These discussions will be audio-recorded and transcribed alongside the photographs themselves. For body-mapping, participants' visual representations will be documented through high-resolution photography and accompanied by participant narratives explaining their choices, which will also be audio-recorded and transcribed. The visual data (photographs and body maps) will be analysed alongside the transcripts, with attention to both the visual content and participants' interpretations of their own work.
>
> The yarning circles and individual yarns will be audio-recorded (with informed consent) and transcribed. Yarning will be led by Indigenous researchers and will follow culturally appropriate protocols, including allowing conversations to flow naturally rather than adhering to a rigid interview schedule. All transcribed data from arts-based methods and yarning will be incorporated into the inductive content analysis described above, alongside data from interviews and focus groups, ensuring that all forms of data contribute equally to the findings."

e. Methods and Analysis: p.14, 3.3.3: Please clarify what will you do with the data collected about what helps/hinders implementation. For example, is it your intention to revise the implementation strategies as a result of your findings?

Response:

The data will be used to refine our implementation strategy and to inform strategies for other projects.

This is now noted at the end of Section 3.3.3:

> "The resources will be rolled out nationally, facilitated by the Investigators and Advisory Board, with the help of their wide and diverse networks. We will conduct an Intervention Scalability Assessment[51] with 30-40 stakeholders at inclusive workshops across all Australian States and Territories to identify factors that help or hinder implementation and use of the resources. The implementation strategy will be revised in line with these findings, as needed."

VERSION 2 - REVIEW

Reviewer 1

Name Kelly, Dervla

Affiliation University of Galway College of Medicine Nursing and Health Sciences, School of Pharmacy and Medical Sciences

Date 18-Jun-2026

COI

Thank you for the detailed and thorough responses.

Reviewer 2

Name Tilley, Elizabeth

Affiliation The Open University, School of Health, Wellbeing and Social Care

Date 16-Jun-2026

COI

Thank you for addressing all the reviewer comments so thoroughly.
