# Supplementary material for: Building genetic healthcare together: an Australian co-production three-phase mixed-methods research protocol with people with intellectual disability
Source: BMJ Open. 2026 Jul 10;16(7):e110086. doi: 10.1136/bmjopen-2025-110086 (PMC13358344; doi:10.1136/bmjopen-2025-110086)
Supplement: Author's manuscript [file bmjopen-2025-110086.draft_revisions.pdf]

BMJ Open

BMJ Open is committed to open peer review. As part of this commitment we make the peer review history of every article we publish publicly available.

When an article is published we post the peer reviewers' comments and the authors' responses online. We also post the versions of the paper that were used during peer review. These are the versions that the peer review comments apply to.

The versions of the paper that follow are the versions that were submitted during the peer review process. They are not the versions of record or the final published versions. They should not be cited or distributed as the published version of this manuscript.

BMJ Open is an open access journal and the full, final, typeset and author-corrected version of record of the manuscript is available on our site with no access controls, subscription charges or pay-per-view fees (http://bmjopen.bmj.com).

If you have any questions on BMJ Open's open peer review process please email info.bmjopen@bmj.com

BMJ Open

# Developing inclusive genetic healthcare for people with intellectual disability: A co-production research protocol



| | |
|---|---|
| Journal: | *BMJ Open* |
| Manuscript ID | bmjopen-2025-110086 |
| Article Type: | Protocol |
| Date Submitted by the Author: | 29-Aug-2025 |





| | |
|---|---|
| Complete List of Authors: | Strnadová, Iva; University of New South Wales, School of Education; University of New South Wales Disability Innovation Institute; Self Advocacy Sydney<br>Tso, Michelle; University of New South Wales, School of Education<br>Loblinzk Refalo, Julie ; University of New South Wales, School of Education; Self Advocacy Sydney<br>Roberts, Natalie; University of New South Wales, Discipline of Paediatrics and Child Health, School of Clinical Medicine, Faculty of Medicine and Health<br>Danker, Joanne ; University of New South Wales, School of Education<br>Sarfaraz, Skie ; Self Advocacy Sydney<br>Boyle, Jackie; Genetics of learning disability service; University of New South Wales, Discipline of Paediatrics and Child Health, Faculty of Medicine and Health<br>Terrill, Bronwyn ; Australian Genomics; University of New South Wales, Faculty of Medicine and Health; Garvan Institute of Medical Research<br>Halliburton, Celia ; NSW Ministry of Health<br>Pantoja Mardones, Claudia ; University of New South Wales, School of Education<br>Hayes, Sarah ; University of New South Wales, School of Education<br>Hurd, Sam ; University of New South Wales, School of Education<br>King, Caitlin; Central Queensland University<br>Barlow-Stewart, Kristine; University of Sydney, Northern Clinical School<br>Evans, Elizabeth ; University of New South Wales, Department of Developmental Disability<br>Leonard, Helen ; The University of Western Australia - Perth Campus; The Kids Research Institute Australia<br>Mar Fan, Helen ; Icon Cancer Centre Auchenflower<br>Rodgers, Jonathan ; Genetic Health Queensland<br>Vella, Yvette ; Northern Sydney Local Health District<br>Mcgaughran, Julie ; Genetic Health Queensland<br>Best, Stephanie ; University of Melbourne<br>Turbitt, Erin; University of Technology Sydney<br>Leach Scully, Jackie ; University of New South Wales Disability Innovation Institute<br>Pratt, Greg; Central Queensland University, Mental Health and Genomics<br>Palmer, Elizabeth; University of New South Wales; Sydney Children's Hospitals Network Randwick, Clinical Genetics |

| | |
|---|---|
| Keywords: | Health Equity, GENETICS, MEDICAL EDUCATION & TRAINING, Developmental neurology & neurodisability < PAEDIATRICS, Australian Aboriginal and Torres Strait Islander Peoples, Health Literacy |
| | |

SCHOLARONE MANUSCRIPTS

**PROTOCOL BMJ OPEN**

# Developing inclusive genetic healthcare for people with intellectual disability: A co-production research protocol

Running title:

**Inclusive genetic healthcare co-protocol protocol**

For peer review only


## Authors

| Name | Affiliation | ORCID ID |
|---|---|---|
| Iva Strnadová | School of Education, UNSW Sydney, Australia<br>Disability Innovation Institute, UNSW Sydney, Australia<br>Self Advocacy Sydney Inc., Australia | 0000-0002-8513-5400 |
| Michelle Tso | School of Education, UNSW Sydney, Australia | 0000-0003-0134-1053 |
| Julie Loblinzk Refalo | School of Education, UNSW Sydney<br>Self Advocacy Sydney Inc. , Australia | 0000-0003-1350-6089 |
| Natalie Roberts | Discipline of Paediatrics and Child Health, School of Clinical Medicine, Faculty of Medicine and Health, UNSW Sydney, Australia | 0009-0000-9906-4589 |
| Joanne Danker | School of Education, UNSW Sydney, Australia | 0000-0001-9071-1903 |
| Skie Sarfaraz | Self Advocacy Sydney Inc., Australia | 0000-0001-5895-8551 |
| Jackie Boyle | Genetics of Learning Disability Service, Waratah, NSW, Australia | 0000-0002-9943-8955 |
| Bronwyn Terrill | Australian Genomics, Melbourne, Australia<br>St Vincent’s Healthcare Clinical Campus, School of Clinical Medicine, Faculty of Medicine and Health, UNSW Sydney, Australia<br>Centre for Population Genomics, Garvan Institute of Medical Research, Darlinghurst, Australia | 0000-0003-0263-363X |


For peer review only - http://bmjopen.bmj.com/site/about/guidelines.xhtml

| Name | Affiliation | ORCID ID |
|---|---|---|
| Celia Halliburton | NSW Ministry of Health, Reserve Road, St Leonards, NSW, Australia | |
| Claudia Pantoja Mardones | School of Education, UNSW Sydney, Australia | 0009-0005-8914-8101 |
| Sarah Hayes | School of Education, UNSW Sydney, Australia | |
| Sam Hurd | School of Education, UNSW Sydney, Australia | |
| Caitlin King | Central Queensland University, Brisbane, Queensland, Australia | |
| Kristine Barlow-Stewart | Northern Clinical School, Faculty of Medicine and Health, University of Sydney, NSW, Australia | 0000-0003-1831-9944 |
| Elizabeth Evans | Department of Developmental Disability Neuropsychiatry, UNSW Sydney; Psychologist in private practice, Sydney, NSW, Australia. | 0000-0002-9046-9501 |
| Helen Leonard | The University of Western Australia, Perth, WA, Australia<br>The Kids Research Institute, Western Australia, Australia | 0000-0001-6405-5834 |
| Helen Mar Fan | Genetic Health Queensland, Brisbane, Queensland | 0000-0002-0267-4047 |
| Jonathan Rodgers | Genetic Health Queensland, Brisbane, Queensland | 0000-0003-0760-8943 |
| Yvette Vella | Primary and Community Health, Northern Sydney Local Health District, NSW, Australia | 0009-0007-1423-5061 |
| Julie McGaughran | Genetic Health Queensland, University of Queensland School of Medicine, Brisbane, Qld, Australia | 0000-0002-0926-7858 |
| Stephanie Best | University of Melbourne, Melbourne, Vic, Australia | 0000-0002-1107-8976 |
| Erin Turbitt | University of Technology, Sydney, Ultimo, NSW | 0000-0002-6650-9702 |
| Jackie Leach Scully | Disability Innovation Institute, UNSW Sydney, NSW, Australia | 0000-0002-7637-1225 |




For peer review only - http://bmjopen.bmj.com/site/about/guidelines.xhtml

| Name | Affiliation | ORCID ID |
| --- | --- | --- |
| Greg Pratt | Central Queensland University, Brisbane, Queensland, Australia | 0000-0001-9908-773X |
| Elizabeth Emma Palmer | Faculty of Medicine and Health, UNSW Sydney; Sydney Children's Hospitals Network, Sydney, NSW, Australia | 0000-0003-1844-215X |

**Corresponding author**

Elizabeth Emma Palmer

Discipline of Pediatrics and Child Health,

School of Clinical Medicine, Faculty of Medicine and Health,

Bright Alliance Building, Level 8,

University of New South Wales,

Randwick, NSW, Australia.

Ph: +61 02 9348 1601

E: Elizabeth.palmer@unsw.edu.au


For peer review only

## ABSTRACT

**Introduction** People with intellectual disability face significant barriers accessing genetic healthcare, including during appointments. Many adults with intellectual disability are denied opportunities to discuss genetic testing that could improve their health and wellbeing. This study follows best practice in inclusive research, with co-researchers with intellectual disability co-leading alongside educational, disability and clinical experts to co-design and implement a respectful, accessible, and inclusive national model of genetic healthcare**.**

**Methods and analysis** This three-phase qualitative study positions people with intellectual disability as active researchers and partners throughout. Phase 1 will engage approximately 100 participants - people with intellectual disability (with and without genetic testing experience), families/support people, and genetic healthcare professionals - through interviews, focus groups, arts-based methods, and yarning circles. Phase 2 will co-design Guiding Principles. Phase 3 will develop practical resources and investigate national roll-out using workshops across Australia, guided by three Community Engagement Groups including people with intellectual disability, families/support people, and healthcare providers. Aboriginal and Torres Strait Islander research methods will be integrated throughout, with Indigenous researchers leading these components. The study will identify barriers and enablers to inclusive genetic healthcare, co-designing Guiding Principles, resources, and implementation recommendations.

**Ethics and Dissemination** Ethics approval has been granted by UNSW Sydney and the Australian Institute of Aboriginal and Torres Strait Islander Studies. All participants will provide written consent using Easy Read materials as appropriate. Findings will be disseminated through journal articles, conferences, websites, and accessible formats including Easy Read summaries and videos**.**


**Registration details (if appropriate)** Not applicable.

For peer review only

## Strengths and limitations of this study

### Strengths

- Authentic inclusive research methodology with people with intellectual disability as co-researchers and co-leaders throughout all phases
- Multi-modal data collection methods (interviews, focus groups, arts-based approaches), including Aboriginal and Torres Strait Islander research methodologies (yarning circles) with Indigenous researcher leadership
- Sequential three-phase design ensures participant involvement from research conception through to national implementation planning

### Limitations

- Convenience sampling approach may limit generalizability of findings despite efforts to recruit across age, geography, support needs, and genetic testing experience
- Resource constraints may limit depth of engagement possible in remote areas or with specific population subgroups

### Manuscript details

| Manuscript | Abstract | Tables | Figures |
|---|---|---|---|
| 2952 words | 248 words | 0 | 2 |

For peer review only

# 1 INTRODUCTION

Intellectual disability refers to neurodevelopmental conditions that begin in childhood, impacting conceptual, social, and practical areas of living.[1] However, each person's specific strengths and support needs are unique. Traditional healthcare typically emphasises limitations rather than capabilities, often excluding people with intellectual disability from decision-making processes.[2] There is compelling evidence that many health professionals exclude people with intellectual disability from consultations, misattribute health problems to their disability, and sometimes display unprofessional behaviour.[3,4] The right to equitable and accessible healthcare, as mandated by the *United Nations Convention on the Rights of Persons with Disabilities (2006)*,[5] is being ignored.

Healthcare workers lack training and confidence in providing healthcare to people with intellectual disability and tend to make assumptions about the capacity of the person to make their own informed choices.[2] They often direct communications to family members, assuming that the person with intellectual disability cannot understand or contribute to healthcare discussions.[6,7] Our work challenges these assumptions by recognising that with appropriate accommodations and respectful engagement, people with intellectual disability can and should be active participants in their healthcare decisions.[8]

People with intellectual disability, especially those with high support needs, experience immense health inequalities, including premature mortality and disproportionate rates of potentially avoidable deaths as a result of late diagnosis or mismanagement.[9-12] People with intellectual disability are frequently neglected and abused within the healthcare system.[13,14]. There is growing awareness of the need for strengths-based, person-centered and trauma-informed healthcare that promotes safety, trust, collaboration, choice and empowerment to ensure that people with intellectual disability are engaged in their own healthcare [15].



For peer review only - http://bmjopen.bmj.com/site/about/guidelines.xhtml

Genetic healthcare is the application of genetic knowledge in diagnosis, prevention, and treatment to improve health outcomes.[22,23] Genetic studies have revolutionised our understanding of the causes of intellectual disability, with over 1,000 genes now implicated.[16,17] Chromosomal microarray, screening for fragile X syndrome, and then more comprehensive genomic testing such as exome or genome sequencing are recommended as diagnostic tests for neurodevelopmental conditions.[17,18] Intellectual disability and/or global developmental delay are the most frequent reasons for medical referral, accounting for 20-30% of genetic testing across all ages and 50-70% in paediatric populations.[19-23] Identifying the genetic basis of a person's intellectual disability has numerous potential benefits. Clinically, it enables surveillance for known complications, guides medical referrals, and informs specific therapies. Individually, it helps people understand their condition, connects them with peer support groups, improves access to appropriate services, informs reproductive choices, and contributes to better quality of life outcomes.[16,24-27]

However, whether people with intellectual disability are fully benefiting from these potential advantages has historically been under-researched. Our recent systematic literature review found only seven studies had been conducted up to 2021 to investigate the opinions and experiences of people with intellectual disability in genetic counselling and testing, highlighting a major gap in knowledge.[28] We also found that few resources on genetics and genetic conditions had been developed specifically for people with intellectual disability, further excluding this group from informed participation in genetic healthcare.[28,29]

The lack of appropriate resources and services is likely to have a disproportionate impact on Australian Aboriginal and Torres Strait Islander people with intellectual disability. The prevalence of intellectual disability in this population is two to three times higher than among non-Indigenous Australians.[30,31] Beyond the common challenges faced by people with intellectual disability, they also encounter layers of institutional and personal

For peer review only

discrimination,[32] use general and genetic health services less frequently than non-Indigenous people, and consistently report being treated in excluding or dismissive ways by healthcare professionals.[13] Strategies to realise the promise of genetic healthcare must address these issues through the development and implementation of culturally appropriate models of genetic healthcare for Aboriginal and Torres Strait Islander people with intellectual disability. This research must be led by Indigenous researchers in partnership with Indigenous people, families, professionals and communities.[33]

Our initial Australian pilot study interviewed adults with intellectual disability who had previous contact with genetics services.[34] The findings painted a concerning picture: participants reported often feeling excluded and unsupported, experiencing anxiety and depression as a result. We identified numerous missed opportunities for genetic diagnoses that could have informed practical healthcare plans, disability supports, and therapies. The study highlighted the urgent need for new approaches and for expanding our research to include people with higher support needs, those from different regions, family members and support people, and clinicians themselves.[8,34]

## 2 PROJECT AIMS

The GeneEQUAL research program aims to develop and evaluate an inclusive, person-centred, and respectful model of genetic healthcare for people with intellectual disability, that addresses the limitations and inequities in the current provision. Our three main aims are:

1. **To understand what prevents and enables access to and provision of inclusive genetic healthcare**. We aim to identify barriers and enablers from the perspectives of people with intellectual disability across all support needs, including Aboriginal and



For peer review only - http://bmjopen.bmj.com/site/about/guidelines.xhtml

Torres Strait Islander people, their families and support people, and healthcare professionals.

2. **To co-design evidence-based solutions.** We aim to develop Guiding Principles that healthcare services can use to overcome barriers and create inclusive, person-centred and respectful genetic healthcare for all people with intellectual disability.
3. **To create resources for real-world implementation.** We aim to co-produce a suite of resources and implementation strategies that enable services to deliver best practice in genetic healthcare nationally and globally.

Across these aims, we are committed to addressing the challenges faced by Aboriginal and Torres Strait Islander people with intellectual disability, ensuring our approach is culturally safe and appropriate.

For peer review only



For peer review only - http://bmjopen.bmj.com/site/about/guidelines.xhtml

# 3 METHODS AND ANALYSIS

## 3.1 Theoretical Framework and Inclusive Design

This project combines social constructivist epistemology and critical realist ontology to support meaningful co-production with people with intellectual disability.[35] Our approach recognises that while genetic conditions have a biological basis,[36,37] their impact is shaped by social and cultural factors.[37,38] The centering of co-production aligns with our commitment to doing research *with* people with intellectual disability rather than *on* them.

The study adheres to the principles described in “Doing Research Inclusively: Co-Production in Action”,[28] ensuring all stakeholders have meaningful roles in knowledge creation. Three Community Engagement Groups (CEGs), representing people with intellectual disability, families/support people, and healthcare providers, will actively shape all research. The CEGs will have membership ensuring representation across age, gender, Aboriginal and Torres Strait Islander heritage, ethnicity, and locality (i.e. regional, remote, rural and metropolitan).

## 3.2 Engagement Structure

Our engagement structure includes overlapping groups to ensure rich and varied perspectives (Figure 1). The GeneEQUAL Advisory Board includes CEG representatives, government and professional organisation members, disability advocacy and support groups, experts in trauma-informed care, and an Aboriginal and Torres Strait Islander Research group. The Guiding Principles Development Group (GPDG) includes members with intellectual disability, family members, healthcare workers and researchers.

### 3.2.1 Patient and public involvement



For peer review only - http://bmjopen.bmj.com/site/about/guidelines.xhtml

People with intellectual disability and their families/support people will be included from the beginning of this study. The research questions and project plan are informed by the priorities, experiences and preferences identified in previous work[8,34] and by GeneEQUAL's research team. GeneEQUAL involves people with intellectual disability as chief and associate investigators and advisory board members, with key roles in co-designing and co-leading the study. GeneEQUAL builds on a decade-long research partnership between Professor Iva Strnadová and Julie Loblinzk Refalo OAM, a self-advocacy leader and researcher with intellectual disability. This collaborative relationship has evolved into authentic co-leadership across 22 funded projects. Julie and Iva have also co-mentored other people with intellectual disability into research roles, creating a cascading model where experienced researchers with intellectual disability guide newcomers. This demonstrates that meaningful co-production emerges from sustained relationships, mutual respect, and genuinely shared research leadership.

People with intellectual disability will help to choose outcome measures and will be essential in participant recruitment. They will play key roles in developing the dissemination plans, including Easy Read summaries, short videos, and accessible newsletters.

### 3.3 Project Design

Our previous work, co-designing the GeneEQUAL Educational Toolkit in the state of New South Wales with people with intellectual disability[8] informed the design of this current study. Our rationale and assumptions in designing this program were based on this previous successful work.

This three-phase qualitative study progresses from understanding to action to implementation (Figure 2). Each phase builds upon previous findings while maintaining the continuous involvement of people with intellectual disability.


For peer review only - http://bmjopen.bmj.com/site/about/guidelines.xhtml

### 3.3.1 Phase 1: Exploring Barriers and Enablers to Inclusive Genetic Healthcare

We will recruit four groups totalling approximately 100 people to participate in qualitative research to help us explore in depth barriers and enablers to inclusive, person-centred and respectful genetic healthcare. The groups will be: 1. People with intellectual disability with genetic testing and/ or counselling experience, 2. People with intellectual disability without genetic testing and/or counselling experience, 3. Family members/support people, and 4. Healthcare professionals. We will use convenience sampling but will strive to maximise diversity across age, gender, ethnicity, and location.

We will offer flexible and accessible ways to participate, including interviews or focus groups (online or in person), in person arts-based methods (Photo-voice, body-mapping)[39,40], and yarning circles for Aboriginal and Torres Strait Islander participants. Yarning in groups (i.e. a yarning circle) or in one-on-one conversations (i.e. "a yarn") are a recognised qualitative research method and offer an alternative to Aboriginal and/or Torres Strait Islander people who prefer these.[41,42] We estimate that interviews and focus groups will last 30-60 minutes; they will be audio-recorded (with informed consent) and transcribed. Interview and focus group protocols will be co-designed through an iterative process, drawing on literature review findings, previous GeneEQUAL studies, and collaborative input from research team experts including researchers with intellectual disability, family members/support people, and healthcare professionals. This ensures protocols are both academically grounded and experientially relevant. The full interview and focus group protocols are available from the corresponding author.

We will use inductive content analysis, a qualitative method well-suited to exploratory research.[43] The analysis will follow a five-step process: (1) familiarisation through thorough reading of all transcripts, (2) open coding to identify units of meaning, (3)



For peer review only - http://bmjopen.bmj.com/site/about/guidelines.xhtml

category development, where categories and subcategories are developed in parallel through iterative refinement of the codes, (4) comparison and refinement of all categories and subcategories, and (5) thematic abstraction to develop overarching themes and subthemes that synthesise the categories that can answer our research questions.[44]

To enhance rigour, multiple researchers will code independently, followed by meetings to reach consensus on data organisation.[45] Field notes and mind-maps will be used to prompt data analysis.[46] Regular team discussions and participant validation will ensure findings remain grounded in participants' experiences.

### 3.3.2 Phase 2: Co-designing Guiding Principles for Inclusive Genetic Healthcare

The Guiding Principles Development Group (GPDG) will be guided by Phase 1 findings  Development and evaluation will follow the Appraisal Guidelines for Research and Evaluation (AGREE) II-compliant processes,[47] using the Grading of Recommendations, Assessment, Development and Evaluation (GRADE) framework.[48] The draft principles will be evaluated by expert review and consultations. When analysing the collected feedback, we will use researcher triangulation (where several team members independently review and code the same data) and member checking (where participants confirm whether our interpretations of the data accurately reflect their perspectives) to ensure consistency and reliability.

To ensure the national Guiding Principles are a ‘living document’, regular biannual meetings of the GPDG will be held throughout the project to update the Guiding Principles, guided by our Advisory Board and CEGs.



For peer review only - http://bmjopen.bmj.com/site/about/guidelines.xhtml

### 3.3.3 Phase 3: Co-develop a Suite of Resources for Implementation of Best Practice

Using our pilot study findings and stakeholder input, we will co-develop a suite of resources including educational materials such as videos, Easy Read social stories, and fact sheets; communication tools for people with high support needs; and training resources for genetic healthcare professionals. CEG workshops will guide our decisions about content, media, and design preferences. We will evaluate the impact of the resources through online surveys and interviews, using adapted Patient Activation Measure and Genomics Outcome Scale items. [49,50] Surveys conducted during the intervention will explore whether healthcare professionals' capability, motivation and opportunity to apply best practice have changed.[8]

We will conduct an Intervention Scalability Assessment[51] with 30-40 stakeholders at inclusive workshops across all Australian States and Territories to identify factors that help or hinder implementation.

## 3.4 Sample Size and Data Saturation

Our sample size is based on established principles for qualitative research.[52] We aim for approximately 100 participants across the four stakeholder groups, with around 25 people per group. Recent systematic reviews show that qualitative research consistently reaches a point defined as saturation within 9-17 interviews or 4-8 focus group discussions, particularly for studies with similar populations and clearly defined objectives.[53,54] Data saturation, the point when new data become redundant and repeat what was previously expressed, is particularly relevant to inductive content analytic approaches.[43] Our pilot study with 15 participants with intellectual disability reached data saturation, which supports our proposed sample size.[34] Applying these principles to data collection: if new data repeat what was expressed in previous data, with no new content categories or themes emerging after 3 additional interviews, we will stop recruitment for that group. However, if new themes or content



For peer review only - http://bmjopen.bmj.com/site/about/guidelines.xhtml

categories are identified, we will extend recruitment up to 35 per group to ensure comprehensive coverage.[53]

# 4 ETHICS AND DISSEMINATION

## 4.1 Ethics Approvals

Ethics approval for Phase 1 was granted by the UNSW Sydney Human Research Ethics Committee (HC230353) and the Australian Institute of Aboriginal and Torres Strait Islander Studies (AIATSIS; REC-0284). Our approach respects Indigenous data sovereignty principles, with Indigenous leadership guiding how information is collected, interpreted, and shared. Ethics approval for Phases 2 and 3 is in process.

## 4.2 Informed consent

We use a voluntary written informed consent process for all participants, including those with intellectual disability. This approach is based on extensive feedback from people with intellectual disability, that assuming incapacity to consent is offensive and risks triggering post-traumatic stress from previous experiences where they were required to prove their capacity.[8] The project team includes co-researchers with intellectual disability, reflecting our commitment to people with intellectual disability being active participants in research decisions. We will use a continuous consent process and include reasonable adjustments such as extended time, and Easy Read participant information sheets and consent forms. We will use the Teach-Back method to ensure understanding, where participants explain information they have been given in their own words, researchers clarify any misunderstandings, and this process continues until comprehension is achieved.[55] Participants will be encouraged to bring someone they trust to interviews, and will be assured that they can withdraw at any time without affecting their relationship with researchers.



For peer review only - http://bmjopen.bmj.com/site/about/guidelines.xhtml

### 4.3 Data Management

Data will be audio-recorded and transcribed by the research team, with all identifiers removed. Pseudonyms will replace names and the master de-identification list will be stored separately from anonymised data on secure, password-protected systems accessible only to team members.

### 4.4. Safety Monitoring

The research team is experienced in interviewing people with intellectual disability and will manage any participant distress, arrange appropriate support if needed through family members, support workers, or general practitioners. We will implement measures to support self-care and researcher safety and well-being, to build researcher resilience and prevent vicarious trauma.[56]

### 4.4 Dissemination Plan

All materials will be available on our accessible website (www.GeneEQUAL.com). Findings will be disseminated through peer-reviewed publications, conference presentations, health professional and self-advocacy websites, and social media. Participants will receive accessible summaries of findings via email or video, with researchers available to answer questions as necessary.

## 5 DISCUSSION

The GeneEQUAL project addresses a critical gap in healthcare equity by developing the world's first inclusive, person-centred genetic healthcare model for people with intellectual



For peer review only - http://bmjopen.bmj.com/site/about/guidelines.xhtml

disability. Our pilot research revealed that people with intellectual disability frequently feel unsupported in genetic healthcare, with numerous missed opportunities for obtaining genetic diagnoses that could inform practical healthcare plans. This protocol outlines our approach to transforming this situation through rigorous co-production methodologies.

The strength of our approach lies in genuine co-production, where people with intellectual disability contribute as active researchers rather than passive subjects. Our three-phase design deliberately moves from understanding barriers, to developing and then implementing solutions. Using different data collection methods (including arts-based approaches [39,57] and yarning circles,[58]) allows people to participate in line with their communication preferences and ways of being, knowing and doing.[59] The resulting Guiding Principles will be distinctive in having emerged directly from lived experience rather than from professional assumptions of what people need.

We anticipate several practical outcomes. For people with intellectual disability, the resources will support meaningful participation in healthcare decisions. For their families and support people it will enable accessible and strengths-based communication about genetics. For healthcare professionals, the project will provide evidence-based approaches to inclusive healthcare. For healthcare systems, examining how to implement a more inclusive model of genetic healthcare will identify pathways for scaling these approaches nationally.

A central challenge is that meaningful co-production is resource-intensive and requires sustained commitment from all stakeholders. Our layered engagement structure aims to address this by distributing responsibility across the CEGs. Additionally, the heterogeneity of intellectual disability means resources must be adaptable to different support needs and cultural contexts, which our emphasis on multimodal resources and iterative testing aims to address.



For peer review only - http://bmjopen.bmj.com/site/about/guidelines.xhtml

While focused on Australia, this work has broad international relevance. The exclusion of people with intellectual disability from advances in genetic healthcare is a global phenomenon. Our methodological approach offers a template that could be adapted to other healthcare systems and may also inform co-production approaches in other clinical fields where people with intellectual disability face similar barriers to healthcare participation.

In the long term**,** this research will foster a paradigm shift from health research that is done *on* people with intellectual disability to that which *is led by* people with intellectual disability. It can also serve as a model for other areas of health, given the urgent need for radical improvement in all healthcare for people with intellectual disability. The mentoring of co-researchers through our program will also grow the next generation of researchers with intellectual disability.

For peer review only


For peer review only - http://bmjopen.bmj.com/site/about/guidelines.xhtml

**Authors contributions**

IS, EEP, JLR and JLS conceived the idea of the project. IS, JLR, EEP, JLS, GP, ET, SB, SS and JM developed the study concept and initiated the project intervention. IS, MS and EEP led the development of an in-depth protocol. EEP, IS, MT and NR wrote the manuscript. All authors reviewed and provided input into the manuscript and read and approved the final version.

**Funding statement**

This work was supported by the Australian National Health & Medical Research Council (NHMRC) Targeted Call for Research into Improving Health of People with Intellectual disability. Research grant title "GeneEQUAL: equitable and accessible genomic healthcare for people with intellectual disability". NHMRC application ID: 2022/GNT2015753. EEP is supported by a National Health and Medical Research Council investigator grant (2021/GNT2008166)

**Competing interests**

The authors declare the following competing interests. IS, JLR, JD, JM, SB, ET, JLS, GP and EEP have received funding to their institutions from the NHMRC. MT, SS, CPM, SH, SH, CK have received funding for research assistance from the NHMRC. JB has received funding for a PhD Scholarship from the NHMRC. KBS has received consultancy fees from Belkin Consulting and payment for honoraria for conference and support for travel from UNIKOM Bandung Indonesia. EEP has received funding to attend meetings from the national peak body for rare diseases Rare Voices Australia.

For peer review only

**Acknowledgements**

The authors acknowledge the GeneEQUAL Advisory Board (in alphabetical order): Yemima Berman, Donna Best, Aimee Blackam, Jasmine Bojadzieva, Emma Bonser, Tiffany Boughtwood, Marie Brand, Mary Butterworth, Emily Caska, Samantha Cooms, Emily Cukalevski, Rachael Cvejic, Dalal Dawood Baumgartner, Leon Elman, Anna Flynn, Emma Gibson, Sally Heggaton, Falak Helwani, Paul Jarnet, Nick Lennox, Trinity Mahede, Belinda McClaren, Cassandra McDonald, Saras Menon, Cathy Milne, Laura Naing, Mary O'Donovan, Trevor Parmenter, Trupti Prasad, Thulasee Sri Ganeshan, Tracey Szanto, Georgina Schlub, Maree Toombs, Michelle Ward

For peer review only

## REFERENCES

1. American Psychiatric Association. Diagnostic and statistical manual of mental disorders (DSM-5®). https://doi/book/10.1176/appi.books.97808904255962013 2013.
2. Dunn M, Strnadova I, Scully JL, *et al.* Equitable and accessible informed healthcare consent process for people with intellectual disability: a systematic literature review. *BMJ Qual Saf* 2024;33:328-39.
3. Ali A, Scior K, Ratti V, *et al.* Discrimination and other barriers to accessing health care: perspectives of patients with mild and moderate intellectual disability and their carers. *PLoS ONE* 2013;8:e70855.
4. Mimmo L, Harrison R, Hinchcliff R. Patient safety vulnerabilities for children with intellectual disability in hospital: a systematic review and narrative synthesis. *BMJ Paediatr Open* 2018;2:e000201.
5. United Nations. Convention on The Rights of Persons with Disabilities (A/RES/61/106). https://social.desa.un.org/issues/disability/crpd/convention-on-the-rights-of-persons-with-disabilities-crpd. 2006.
6. Molnar C, Strnadova I, Dunn M, *et al.* The need for co-educators to drive a new model of inclusive, person-centred and respectful co-healthcare with people with intellectual disability. *Front Psychiatry* 2024;15:1346423.
7. Brooker KS, De Greef R, Trollor JN, *et al.* Intellectual disability healthcare in Australia: Progress, challenges, and future directions. *Journal of Policy and Practice in Intellectual Disabilities* 2024;21:e12497.
8. Strnadova I, Dunn M, Molnar C, *et al.* "All doctors should be trained in that": The co-production and mixed-methods evaluation of an educational toolkit to enable safe, high-quality genetic healthcare for people with intellectual disability. *Genet Med* 2025:101371.

9. Trollor J, Srasuebkul P, Xu H, Howlett S. Cause of death and potentially avoidable deaths in Australian adults with intellectual disability using retrospective linked data. *BMJ Open* 2017;7:e013489.
10. Walmsley J. Investigating premature deaths of people with intellectual disabilities: who is protecting whom? *Disabil Soc* 2020;35:1018-23.
11. El Ansari W, Stock C. Is the Health and Wellbeing of University Students Associated with their Academic Performance? Cross Sectional Findings from the United Kingdom. 2010;7:509-27.
12. Bourke J, Nembhard WN, Wong K, Leonard H. Twenty-Five Year Survival of Children with Intellectual Disability in Western Australia. *J Pediatr* 2017;188:232-9 e2.
13. Australian Government Royal Commission into Violence Abuse Neglect and Exploitation of People with Disability. Final Report - Volume 3, Nature and extent of violence, abuse, neglect and exploitation. https://disability.royalcommission.gov.au/publications/final-report-volume-3-nature-and-extent-violence-abuse-neglect-and-exploitation. 2023.
14. Hammarlund M, Granqvist P, Forslund T. Experiences of Interpersonal Trauma Among Parents With Intellectual Disabilities: A Systematic Review. *Trauma Violence Abuse* 2023;24:2843-62.
15. Australian Government Department of Health Disability and Ageing. National Roadmap for Improving the Health of People with Intellectual Disability. https://www.health.gov.au/our-work/national-roadmap-for-improving-the-health-of-people-with-intellectual-disability. 2021.
16. Vissers LE, Gilissen C, Veltman JA. Genetic studies in intellectual disability and related disorders. *Nat Rev Genet* 2016;17:9-18.
17. Srivastava S, Love-Nichols JA, Dies KA*, et al.* Meta-analysis and multidisciplinary consensus statement: exome sequencing is a first-tier clinical diagnostic test for individuals with neurodevelopmental disorders. *Genetics in Medicine* 2019;21:2413-21.
18. Miller DT, Adam MP, Aradhya S*, et al.* Consensus statement: chromosomal microarray is a first-tier clinical diagnostic test for individuals with developmental disabilities or congenital anomalies. *Am J Hum Genet* 2010;86:749-64.
19. Tan TY, Dillon OJ, Stark Z*, et al.* Diagnostic Impact and Cost-effectiveness of Whole-Exome Sequencing for Ambulant Children With Suspected Monogenic Conditions. *JAMA Pediatr* 2017;171:855-62.
20. Yang Y, Muzny DM, Reid JG*, et al.* Clinical whole-exome sequencing for the diagnosis of mendelian disorders. *N Engl J Med* 2013;369:1502-11.
21. Lee H, Deignan JL, Dorrani N*, et al.* Clinical exome sequencing for genetic identification of rare Mendelian disorders. *JAMA* 2014;312:1880-7.
22. Yang Y, Muzny DM, Xia F*, et al.* Molecular findings among patients referred for clinical whole-exome sequencing. *Jama* 2014;312:1870-9.

For peer review only

23. Retterer K, Juusola J, Cho MT, *et al.* Clinical application of whole-exome sequencing across clinical indications. *Genet Med* 2016;18:696-704.
24. van Karnebeek CDM, Houben RFA, Lafek M, *et al.* The treatable intellectual disability APP www.treatable-id.org: a digital tool to enhance diagnosis & care for rare diseases. *Orphanet J Rare Dis* 2012;7:47.
25. Coulter ME, Miller DT, Harris DJ, *et al.* Chromosomal microarray testing influences medical management. *Genetics in Medicine* 2011;13:770-6.
26. Savatt JM, Myers SM. Genetic testing in neurodevelopmental disorders. *Front Pediatr* 2021;9:526779.
27. Kohler JN, Turbitt E, Biesecker BB. Personal utility in genomic testing: a systematic literature review. *Eur J Hum Genet* 2017;25:662-8.
28. Strnadová I, Dowse L, Garcia-Lee B. Doing Research Inclusively: Co-Production in Action. DIIU UNSW Sydney. https://www.disabilityinnovation.unsw.edu.au/inclusive-research/research-resources 2022.
29. Kotwal H, Fleming J, Barlow-Stewart K, *et al.* Pre-genetics clinic resource evaluation for adults with intellectual disability: The pre-genetics clinic aid. *J Genet Couns* 2020;29:668-77.
30. Australian Institute Of Health and Welfare. Intellectual disability in Australia's Aboriginal and Torres Strait Islander peoples. *J Intellect Dev Disabil* 2007;32:222-5.
31. Bourke J, Sanders R, Jones J, *et al.* Intellectual disability and autism prevalence in Western Australia: impact of the NDIS. *Front Psychiatry* 2024;15:1359505.
32. Hamilton SL, Maslen S, Watkins R, *et al.* 'That thing in his head': Aboriginal and non-Aboriginal Australian caregiver responses to neurodevelopmental disability diagnoses. *Sociol Health Illn* 2020;42:1581-96.
33. Australian Government Department of Health Disability and Ageing. Genomics Australia. https://www.health.gov.au/our-work/genomics-australia.
34. Strnadova I, Loblinzk J, Scully JL, *et al.* "I am not a number!" Opinions and preferences of people with intellectual disability about genetic healthcare. *Eur J Hum Genet* 2023;31:1057-65.
35. Bogna F, Raineri A, Dell G. Critical realism and constructivism: merging research paradigms for a deeper qualitative study. *Qualitative Research in Organizations and Management: An International Journal* 2020;15:461-84.
36. Berryman DR. Ontology, Epistemology, Methodology, and Methods: Information for Librarian Researchers. *Med Ref Serv Q* 2019;38:271-9.
37. Hathcoat JD, Meixner C, Nicholas MC. Ontology and Epistemology. In: Liamputtong P, editor. Handbook of Research Methods in Health Social Sciences. Singapore: Springer Singapore; 2019. p. 99-116.




For peer review only - http://bmjopen.bmj.com/site/about/guidelines.xhtml

38. Spencer R, Pryce JM, Walsh J. Philosophical Approaches to Qualitative Research. In: Leavy P, editor. The Oxford Handbook of Qualitative Research: Oxford University Press; 2020. p. 112-42.
39. Danker J, Strnadova I, Cumming TM. Picture my well-being: Listening to the voices of students with autism spectrum disorder. *Res Dev Disabil* 2019;89:130-40.
40. Leavy P. Method meets art: Arts-based research practice, 2nd ed. New York, NY, US: Guilford Press; 2015. xxi, 328-xxi, p.
41. Kennedy M, Maddox R, Booth K*, et al.* Decolonising qualitative research with respectful, reciprocal, and responsible research practice: a narrative review of the application of Yarning method in qualitative Aboriginal and Torres Strait Islander health research. *International Journal for Equity in Health* 2022;21:134.
42. Lewis T, Hill AE, Bond C, Nelson A. Yarning. *Journal of Clinical Practice in Speech-Language Pathology* 2017;19:14-8.
43. Saunders B, Sim J, Kingstone T*, et al.* Saturation in qualitative research: exploring its conceptualization and operationalization. *Qual Quant* 2018;52:1893-907.
44. Vears D, Gillam L. Inductive content analysis: A guide for beginning qualitative researchers. *Focus on Health Professional Education: A Multi-Professional Journal* 2022;23:111-27.
45. Lovrić R, Farčić N, Mikšić Š, Včev A. Studying During the COVID-19 Pandemic: A Qualitative Inductive Content Analysis of Nursing Students' Perceptions and Experiences. *Education Sciences* 2020;10:188.
46. Cascio MA, Racine E. Person-oriented research ethics: integrating relational and everyday ethics in research. *Account Res* 2018;25:170-97.
47. Brouwers MC, Kho ME, Browman GP*, et al.* AGREE II: advancing guideline development, reporting and evaluation in health care. *CMAJ* 2010;182:E839-42.
48. Prasad M. Introduction to the GRADE tool for rating certainty in evidence and recommendations. *Clinical Epidemiology and Global Health* 2024;25:101484.
49. Muraki E. A Generalized Partial Credit Model: Application of an EM Algorithm. 1992;16:159-76.
50. Grant PE, Pampaka M, Payne K*, et al.* Developing a short-form of the Genetic Counselling Outcome Scale: The Genomics Outcome Scale. *Eur J Med Genet* 2019;62:324-34.
51. Milat A, Lee K, Conte K*, et al.* Intervention Scalability Assessment Tool: A decision support tool for health policy makers and implementers. *Health Res Policy Syst* 2020;18:1.
52. Braun V, Clarke V. To saturate or not to saturate? Questioning data saturation as a useful concept for thematic analysis and sample-size rationales. *Qualitative Research in Sport, Exercise and Health* 2021;13:201-16.
53. Hennink M, Kaiser BN. Sample sizes for saturation in qualitative research: A systematic review of empirical tests. *Soc Sci Med* 2022;292:114523.



For peer review only - http://bmjopen.bmj.com/site/about/guidelines.xhtml

54. Wutich A, Beresford M, Bernard HR. Sample Sizes for 10 Types of Qualitative Data Analysis: An Integrative Review, Empirical Guidance, and Next Steps. *International Journal of Qualitative Methods* 2024;23:16094069241296206.

55. Talevski J, Wong Shee A, Rasmussen B*, et al.* Teach-back: A systematic review of implementation and impacts. *PLoS ONE* 2020;15:e0231350.

56. Smith E, Pooley JA, Holmes L*, et al.* Vicarious Trauma: Exploring the Experiences of Qualitative Researchers Who Study Traumatized Populations. *Disaster Med Public Health Prep* 2021;17:e69.

57. Danker J, Strnadová I, Cumming TM. Engaging Students With Autism Spectrum Disorder in Research Through Participant-Driven Photo-Elicitation Research Technique. *Australasian Journal of Special Education* 2017;41:35-50.

58. Christensen K. Yarning as decolonising practice. *Int J Narrat Ther Community Work* 2022:1-8.

59. Bessarab D. Changing How and What We Do: The Significance of Embedding Aboriginal and Torres Strait Islander Ways of Knowing, Being, and Doing in Social Work Education and Practice. *Australian Social Work* 2015;68:1-4.


## FIGURES

**Figure 1.** Project engagement structure

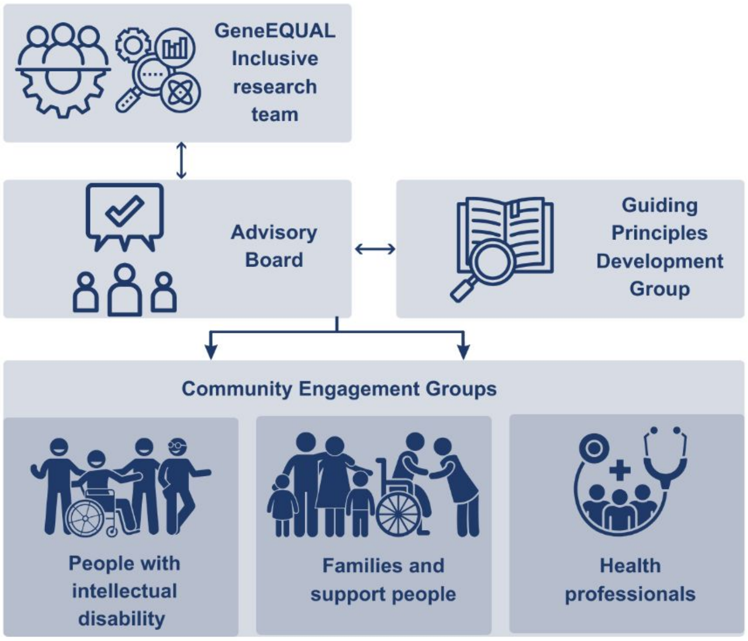




For peer review only - http://bmjopen.bmj.com/site/about/guidelines.xhtml

**Figure 2.** Project phases

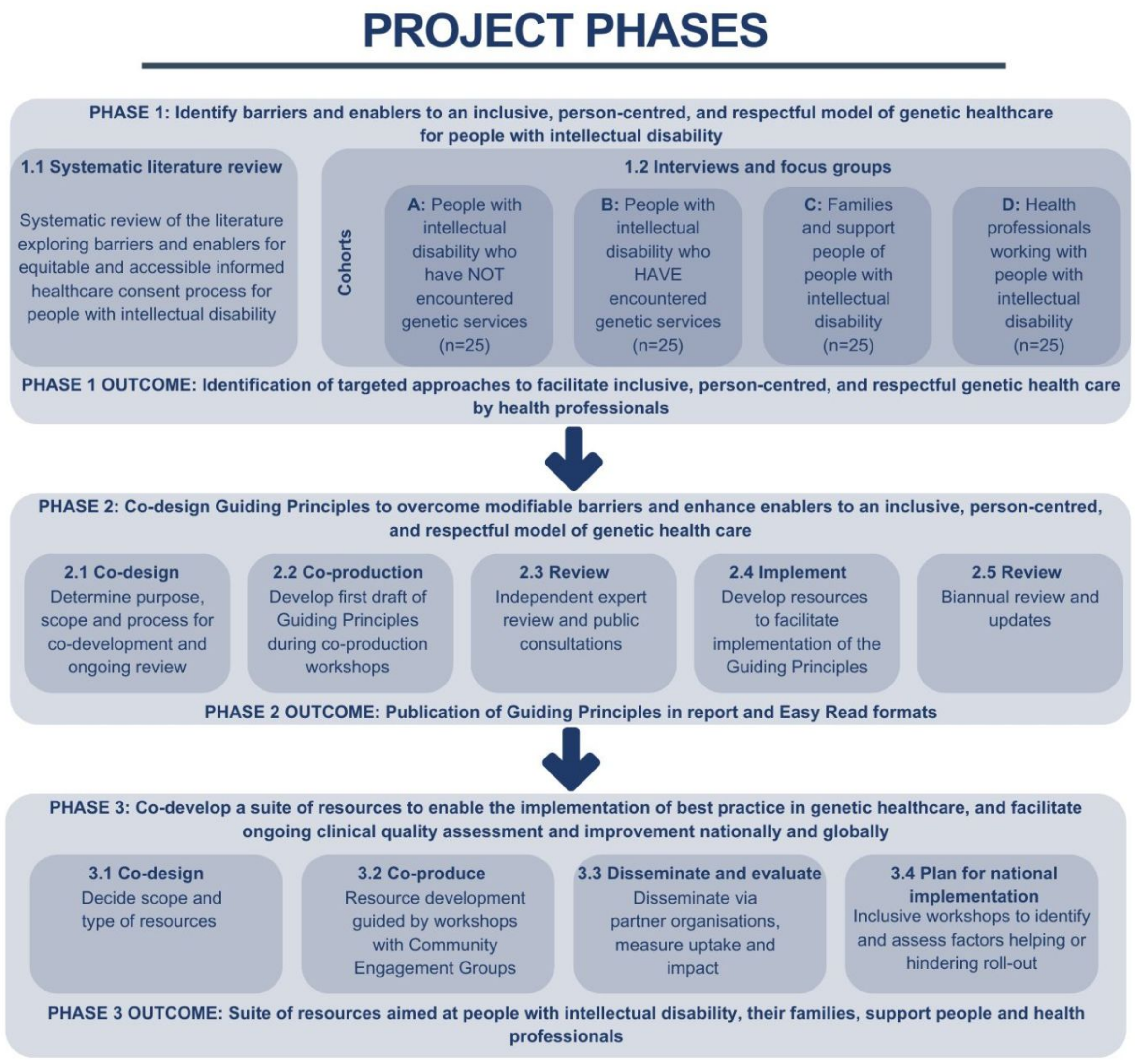

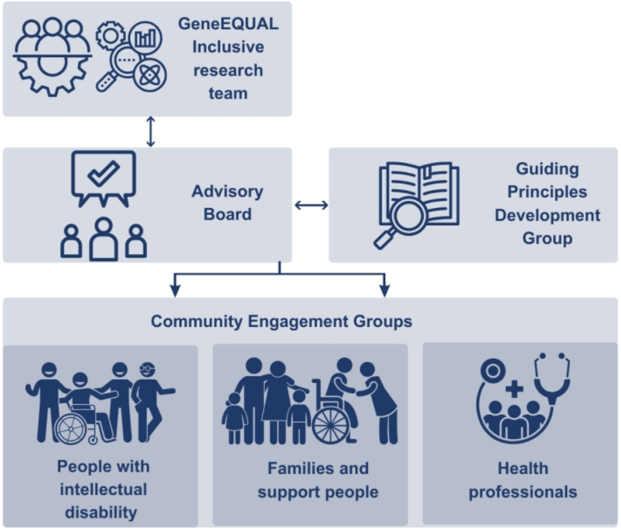


Figure 1. Project engagement structure

758x536mm (67 x 67 DPI)

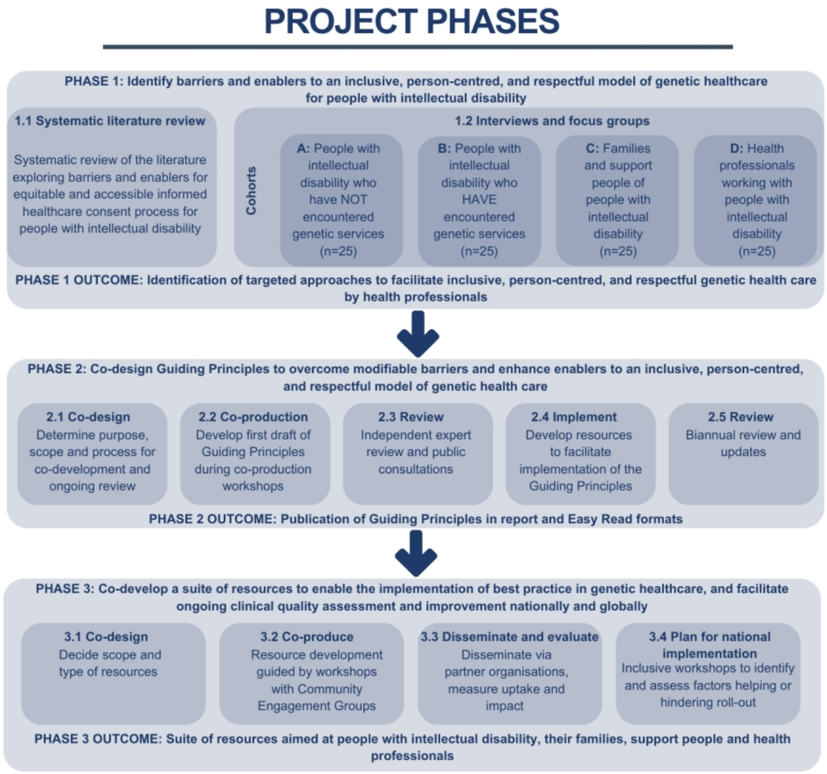


Figure 2. Project phases

536x758mm (67 x 67 DPI)

BMJ Open

# Building genetic healthcare together: An Australian co-production three phase mixed-methods research protocol with people with intellectual disability



| | |
|---|---|
| Journal: | *BMJ Open* |
| Manuscript ID | bmjopen-2025-110086.R1 |
| Article Type: | Protocol |
| Date Submitted by the Author: | 25-May-2026 |





| | |
|---|---|
| Complete List of Authors: | Strnadová, Iva; University of New South Wales, School of Education; University of New South Wales Disability Innovation Institute; Self Advocacy Sydney<br>Tso, Michelle; University of New South Wales, School of Education; University of New South Wales, Discipline of Paediatrics and Child Health, School of Clinical Medicine, Faculty of Medicine and Health,<br>Loblinzk Refalo, Julie ; University of New South Wales, School of Education; Self Advocacy Sydney, Inc.<br>Roberts, Natalie; University of New South Wales, Discipline of Paediatrics and Child Health, School of Clinical Medicine, Faculty of Medicine and Health<br>Danker, Joanne ; University of New South Wales, School of Education<br>Sarfaraz, Skie ; University of New South Wales, School of Education; Self Advocacy Sydney, Inc.<br>Boyle, Jackie; Genetics of learning disability service; University of New South Wales, Discipline of Paediatrics and Child Health, Faculty of Medicine and Health<br>Terrill, Bronwyn ; University of New South Wales, St Vincent's Healthcare Clinical Campus, School of Clinical Medicine, Faculty of Medicine and Health; Garvan Institute of Medical Research, Centre for Population Genomics; Australian Genomics<br>Halliburton, Celia ; NSW Ministry of Health<br>Pantoja Mardones, Claudia ; University of New South Wales, School of Education; University of New South Wales, Discipline of Paediatrics and Child Health, School of Clinical Medicine, Faculty of Medicine and Health<br>Hayes, Sarah ; University of New South Wales, School of Education<br>Hurd, Sam ; University of New South Wales, School of Education<br>King, Caitlin; Central Queensland University<br>Barlow-Stewart, Kristine; University of Sydney, Northern Clinical School<br>Evans, Elizabeth ; University of New South Wales, Department of Developmental Disability Neuropsychiatry<br>Leonard, Helen ; The University of Western Australia - Perth Campus; The Kids Research Institute Australia<br>Mar Fan, Helen ; Genetic Health Queensland<br>Rodgers, Jonathan ; Genetic Health Queensland<br>Vella, Yvette ; Northern Sydney Local Health District, Primary and Community Health<br>Mcgaughran, Julie ; Genetic Health Queensland; The University of Queensland, School of Medicine |

| | |
|---|---|
| | Best, Stephanie ; University of Melbourne<br>Turbitt, Erin; University of Technology Sydney<br>Leach Scully, Jackie ; University of New South Wales Disability Innovation Institute<br>Pratt, Greg; Central Queensland University<br>Palmer, Elizabeth; University of New South Wales, Discipline of Paediatrics and Child Health, School of Clinical Medicine, Faculty of Medicine and Health; Sydney Children's Hospitals Network Randwick, Clinical Genetics |
| <b>Primary Subject Heading</b>: | Genetics and genomics |
| Secondary Subject Heading: | Patient-centred medicine |
| Keywords: | Health Equity, GENETICS, MEDICAL EDUCATION & TRAINING, Developmental neurology & neurodisability < PAEDIATRICS, Australian Aboriginal and Torres Strait Islander Peoples, Health Literacy |
| | |

**PROTOCOL BMJ OPEN**

# Building genetic healthcare together: An Australian co-production three phase mixed-methods research protocol with people with intellectual disability

Running title:

**Inclusive genetic healthcare co-protocol protocol**

For peer review only

## Authors


| Name | Affiliation | ORCID ID |
|---|---|---|
| Iva Strnadová | School of Education, UNSW Sydney, Australia<br>Disability Innovation Institute, UNSW Sydney, Australia<br>Self Advocacy Sydney Inc., Australia | 0000-0002-8513-5400 |
| Michelle Tso | School of Education, UNSW Sydney, Australia<br>Discipline of Paediatrics and Child Health, School of Clinical Medicine, Faculty of Medicine and Health, UNSW Sydney, Australia | 0000-0003-0134-1053 |
| Julie Loblinzk Refalo | School of Education, UNSW Sydney<br>Self Advocacy Sydney Inc., Australia | 0000-0003-1350-6089 |
| Natalie Roberts | Discipline of Paediatrics and Child Health, School of Clinical Medicine, Faculty of Medicine and Health, UNSW Sydney, Australia | 0009-0000-9906-4589 |
| Joanne Danker | School of Education, UNSW Sydney, Australia | 0000-0001-9071-1903 |
| Skie Sarfaraz | School of Education, UNSW Sydney, Australia<br>Self Advocacy Sydney Inc., Australia | 0000-0001-5895-8551 |
| Jackie Boyle | Genetics of Learning Disability Service, Waratah, NSW, Australia<br>Discipline of Paediatrics and Child Health, School of Clinical Medicine, Faculty of Medicine and Health, UNSW Sydney, Australia | 0000-0002-9943-8955 |

| Name | Affiliation | ORCID ID |
|---|---|---|
| Bronwyn Terrill | St Vincent's Healthcare Clinical Campus, School of Clinical Medicine, Faculty of Medicine and Health, UNSW Sydney, Australia<br>Centre for Population Genomics, Garvan Institute of Medical Research, Darlinghurst, Australia<br>Australian Genomics, Melbourne, Australia | 0000-0003-0263-363X |
| Celia Halliburton | NSW Ministry of Health, Reserve Road, St Leonards, NSW, Australia | |
| Claudia Pantoja Mardones | School of Education, UNSW Sydney, Australia<br>Discipline of Paediatrics and Child Health, School of Clinical Medicine, Faculty of Medicine and Health, UNSW Sydney, Australia | 0009-0005-8914-8101 |
| Sarah Hayes | School of Education, UNSW Sydney, Australia | |
| Sam Hurd | School of Education, UNSW Sydney, Australia | |
| Caitlin King | Central Queensland University, Brisbane, Queensland, Australia | |
| Kristine Barlow-Stewart | Northern Clinical School, Faculty of Medicine and Health, University of Sydney, NSW, Australia | 0000-0003-1831-9944 |
| Elizabeth Evans | Department of Developmental Disability Neuropsychiatry, UNSW Sydney;<br>Psychologist in private practice, Sydney, NSW, Australia. | 0000-0002-9046-9501 |
| Helen Leonard | The University of Western Australia, Perth, WA, Australia<br>The Kids Research Institute, Western Australia, Australia | 0000-0001-6405-5834 |
| Helen Mar Fan | Genetic Health Queensland, Brisbane, Queensland | 0000-0002-0267-4047 |
| Jonathan Rodgers | Genetic Health Queensland, Brisbane, Queensland | 0000-0003-0760-8943 |
| Yvette Vella | Primary and Community Health, Northern Sydney Local Health District, NSW, Australia | 0009-0007-1423-5061 |
| Julie McGaughran | Genetic Health Queensland, University of Queensland School of Medicine, Brisbane, Qld, Australia | 0000-0002-0926-7858 |
| Stephanie Best | University of Melbourne, Melbourne, Vic, Australia | 0000-0002-1107-8976 |
| Erin Turbitt | University of Technology, Sydney, Ultimo, NSW | 0000-0002-6650-9702 |



For peer review only

| Name | Affiliation | ORCID ID |
| --- | --- | --- |
| Jackie Leach Scully | Disability Innovation Institute, UNSW Sydney, NSW, Australia | 0000-0002-7637-1225 |
| Greg Pratt | Central Queensland University, Brisbane, Queensland, Australia | 0000-0001-9908-773X |
| Elizabeth Emma Palmer | Faculty of Medicine and Health, UNSW Sydney; Sydney Children's Hospitals Network, Sydney, NSW, Australia | 0000-0003-1844-215X |

**Corresponding author**

Elizabeth Emma Palmer

Discipline of Paediatrics and Child Health,

School of Clinical Medicine, Faculty of Medicine and Health,

Level 3, Health Translation Hub,

Botany Street,

Randwick, NSW, Australia.

Ph: +61 02 9348 1601

E: Elizabeth.palmer@unsw.edu.au


For peer review only

## ABSTRACT

**Introduction** People with intellectual disability face significant barriers accessing genetic healthcare, including during appointments. Many adults with intellectual disability are denied opportunities to discuss genetic testing that could improve their health and wellbeing. This study follows best practice in inclusive research, with co-researchers with intellectual disability co-leading alongside educational, disability and clinical experts to co-design and implement a respectful, accessible, and inclusive national model of genetic healthcare**.**

**Methods and analysis** This three-phase qualitative study positions people with intellectual disability as active researchers and partners throughout. Phase 1 will identify barriers and enablers to genetic healthcare for people with intellectual disability by engaging with approximately 100 participants - people with intellectual disability (with and without genetic testing experience), families/support people, and genetic healthcare professionals - through interviews, focus groups, arts-based methods, and yarning circles. Phase 2 will co-design and evaluate Guiding Principles, providing solutions to identified barriers and leveraging identified facilitators. Phase 3 will develop practical resources using workshops across Australia, guided by three Community Engagement Groups including people with intellectual disability, families/support people, and healthcare providers. This phase will also investigate strategies to optimise national roll-out and implementation of resources among health professionals, people with intellectual disability and their families/carers. Aboriginal and Torres Strait Islander research methods are integrated throughout, led by Indigenous researchers.

**Ethics and Dissemination** Ethics approval has been granted by UNSW Sydney Human Research Ethics Committee (HC230353) and the Australian Institute of Aboriginal and Torres Strait Islander Studies (REC-0284). Participants will provide written consent using Easy Read or plain English materials. Findings will be disseminated through journal articles,


For peer review only

conferences, websites, and accessible formats including Easy Read summaries and videos.**Registration details (if appropriate)** Not applicable.

See the Graphical Abstract for a visual summary.

## Strengths and limitations of this study

### Strengths

- Authentic inclusive research methodology with people with intellectual disability as co-researchers and co-leaders throughout all phases
- Multi-modal data collection methods (interviews, focus groups, arts-based approaches), including Aboriginal and Torres Strait Islander research methodologies (yarning circles) with Indigenous researcher leadership
- Sequential three-phase design ensures participant involvement from research conception through to national implementation planning

### Limitations

- Convenience sampling approach may limit generalisability of findings despite efforts to recruit across age, geography, support needs, and genetic testing experience
- Resource constraints may limit depth of engagement possible in remote areas or with specific population subgroups

## Manuscript details

| **Manuscript** | **Abstract** | **Tables** | **Figures** |
|---|---|---|---|
| 4179 words | 285 words | 0 | 2 |



For peer review only - http://bmjopen.bmj.com/site/about/guidelines.xhtml

## 1 INTRODUCTION

Intellectual disability refers to neurodevelopmental conditions that begin in childhood, impacting conceptual, social, and practical areas of living.[1] However, each person's specific strengths and support needs are unique. Traditional healthcare typically emphasises limitations rather than capabilities, often excluding people with intellectual disability from decision-making processes.[2] There is compelling evidence that many health professionals exclude people with intellectual disability from consultations, misattribute health problems to their disability (known as diagnostic overshadowing), and sometimes display unprofessional behaviour.[3,4] The right to equitable and accessible healthcare, as mandated by the *United Nations Convention on the Rights of Persons with Disabilities (2006)*,[5] is being ignored.

Healthcare workers lack training and confidence in providing healthcare to people with intellectual disability and tend to make assumptions about the capacity of the person to make their own informed choices.[2] They often direct communications to family members, assuming that the person with intellectual disability cannot understand or contribute to healthcare discussions.[6,7] Our work challenges these assumptions by recognising that with appropriate accommodations and respectful engagement, people with intellectual disability can and should be active participants in their healthcare decisions.[8]

People with intellectual disability, especially those with high support needs – that is, people who require extensive or pervasive support across most areas of daily living, which includes many people with severe or profound intellectual disability and/or multiple disabilities – experience immense health inequalities, including premature mortality and disproportionate rates of potentially avoidable deaths as a result of late diagnosis or mismanagement.[9-12] People with intellectual disability are frequently neglected and abused within the healthcare system.[13,14] There is growing awareness of the need for strengths-based,



For peer review only - http://bmjopen.bmj.com/site/about/guidelines.xhtml

person-centered and trauma-informed healthcare that promotes safety, trust, collaboration, choice and empowerment to ensure that people with intellectual disability are engaged in their own healthcare.[15]

Genetic healthcare is the application of genetic knowledge in diagnosis, prevention, and treatment to improve health outcomes.[22,23] Genetic studies have revolutionised our understanding of the causes of intellectual disability, with over 1,000 genes now implicated.[16,17] Chromosomal microarray, screening for fragile X syndrome, and then more comprehensive genomic testing such as exome or genome sequencing are recommended as diagnostic tests for neurodevelopmental conditions.[17,18] Intellectual disability and/or global developmental delay are the most frequent reasons for medical referral, accounting for 20-30% of genetic testing across all ages and 50-70% in paediatric populations.[19-23] Identifying the genetic basis of a person's intellectual disability has numerous potential benefits. Clinically, it enables surveillance for known complications, guides medical referrals, and informs specific therapies. Individually, it helps people understand their condition, connects them with peer support groups, improves access to appropriate services, informs reproductive choices, and contributes to better quality of life outcomes.[16,24-27]

However, whether people with intellectual disability are fully benefiting from these potential advantages has historically been under-researched. Our recent systematic literature review found only seven studies had been conducted up to 2021 to investigate the opinions and experiences of people with intellectual disability in genetic counselling and testing, highlighting a major gap in knowledge.[28] We also found that few resources on genetics and genetic conditions had been developed specifically for people with intellectual disability, further excluding this group from informed participation in genetic healthcare.[28,29]

The lack of appropriate resources and services is likely to have a disproportionate impact on Australian Aboriginal and Torres Strait Islander people with intellectual disability.



For peer review only - http://bmjopen.bmj.com/site/about/guidelines.xhtml

The prevalence of intellectual disability in this population is two to three times higher than among non-Indigenous Australians.[30,31] Beyond the common challenges faced by people with intellectual disability, they also encounter layers of institutional and personal discrimination,[32] use general and genetic health services less frequently than non-Indigenous people, and consistently report being treated in excluding or dismissive ways by healthcare professionals.[13] Strategies to realise the promise of genetic healthcare must address these issues through the development and implementation of culturally appropriate models of genetic healthcare for Aboriginal and Torres Strait Islander people with intellectual disability. This research must be led by Indigenous researchers in partnership with Indigenous people, families, professionals and communities.[33]

Our initial Australian pilot study interviewed adults with intellectual disability who had previous contact with genetics services.[34] The findings painted a concerning picture: participants reported often feeling excluded and unsupported, experiencing anxiety and depression as a result. We identified numerous missed opportunities for genetic diagnoses that could have informed practical healthcare plans, disability supports, and therapies. The study highlighted the urgent need for new approaches and for expanding our research to include people with higher support needs, those from different regions, family members and support people, and clinicians themselves.[8,34]

## 2 PROJECT AIMS

The GeneEQUAL research program aims to develop and evaluate an inclusive, person-centred, and respectful model of genetic healthcare for people with intellectual disability, that addresses the limitations and inequities in the current provision. Our three main aims are:



For peer review only - http://bmjopen.bmj.com/site/about/guidelines.xhtml

1. **To understand what prevents and enables access to and provision of inclusive genetic healthcare**. We aim to identify barriers and enablers from the perspectives of people with intellectual disability across all support needs, including Aboriginal and Torres Strait Islander people, their families and support people, and healthcare professionals.
2. **To co-design evidence-based solutions.** We aim to develop Guiding Principles that healthcare services can use to overcome barriers and create inclusive, person-centred and respectful genetic healthcare for all people with intellectual disability.
3. **To create resources for real-world implementation.** We aim to co-produce a suite of resources and implementation strategies that enable services to deliver best practice in genetic healthcare nationally and globally.

Across these aims, we are committed to addressing the challenges faced by Aboriginal and Torres Strait Islander people with intellectual disability, ensuring our approach is culturally safe and appropriate.

An Easy Read summary of this article is available in the Supplemental Material.

For peer review only

## 3 METHODS AND ANALYSIS

### 3.1 Theoretical Framework and Inclusive Design

This project combines social constructivist epistemology and critical realist ontology to support meaningful co-production with people with intellectual disability.[35] Our approach recognises that while genetic conditions have a biological basis,[36,37] their impact is shaped by social and cultural factors.[37,38] The centering of co-production aligns with our commitment to doing research *with* people with intellectual disability rather than *on* them.

The study adheres to the principles described in "Doing Research Inclusively: Co-Production in Action",[28] ensuring all stakeholders have meaningful roles in knowledge creation. As shown in Figure 1, the three Community Engagement Groups (CEGs) are organised by stakeholder type: (1) people with intellectual disability, (2) families and support people, and (3) health professionals. This structure was chosen to ensure that each group can discuss issues relevant to their specific perspectives and experiences without the potential power imbalances that can arise in mixed stakeholder groups. In particular, separating the group of people with intellectual disability from healthcare professionals and family members ensures that their voices are not overshadowed, and that they can speak freely about their experiences. All three CEGs contribute to the same research phases, and their perspectives are brought together during Advisory Board meetings and in the data analysis process. Each CEG will have at least 6 members and have membership ensuring representation across age, gender, Aboriginal and Torres Strait Islander heritage, ethnicity, and locality (i.e. regional, remote, rural and metropolitan).

The study and Community Engagement Group will include people with intellectual disability across the full range of support needs, from those who live relatively independently with minimal support to those who require extensive or pervasive support in most areas of


For peer review only - http://bmjopen.bmj.com/site/about/guidelines.xhtml

daily living. We use support-needs language rather than clinical severity classifications, consistent with a strengths-based approach that recognises each person's unique requirements. To support the meaningful participation of people with different support needs in the CEGs, we will provide a range of accommodations. These include Easy Read materials for all written documents, visual supports, extended time for discussions, accessible meeting formats (including online and in-person options), co-facilitation by researchers with intellectual disability, and the option for participants to bring a trusted person to meetings. Arts-based methods such as photo-voice and body-mapping will also be offered as alternative ways to contribute for people who prefer non-verbal or creative modes of communication.

The researchers conducting the interviews will be of mixed background and experienced in inclusive research. They include people with intellectual disability, researchers in disability and/or education studies, genomics, genomics education, psychosocial researchers, and teachers.

## 3.2 Engagement Structure

Our engagement structure includes overlapping groups to ensure rich and varied perspectives (Figure 1). The GeneEQUAL Advisory Board is made up of 48 members, including CEG representatives, government and professional organisation members, disability advocacy and support groups, experts in trauma-informed care, and an Aboriginal and Torres Strait Islander Research group. The Guiding Principles Development Group (GPDG) includes members with intellectual disability, family members, healthcare workers and researchers.

All people with intellectual disability will be remunerated via a gift card for their time and expertise in accordance with Australian guidelines.



For peer review only - http://bmjopen.bmj.com/site/about/guidelines.xhtml

3.2.1 Patient and public involvement

People with intellectual disability and their families/support people will be included from the beginning of this study. The research questions and project plan are informed by the priorities, experiences and preferences identified in previous work[8,34] and by GeneEQUAL's research team. GeneEQUAL involves people with intellectual disability as chief and associate investigators and advisory board members, with key roles in co-designing and co-leading the study. GeneEQUAL builds on a decade-long research partnership between Professor Iva Strnadová and Julie Loblinzk Refalo OAM, a self-advocacy leader and researcher with intellectual disability. This collaborative relationship has evolved into authentic co-leadership across 22 funded projects. Julie and Iva have also co-mentored other people with intellectual disability into research roles, creating a cascading model where experienced researchers with intellectual disability guide newcomers. This demonstrates that meaningful co-production emerges from sustained relationships, mutual respect, and genuinely shared research leadership.

People with intellectual disability will help to choose outcome measures and will be essential in participant recruitment. They will play key roles in developing the dissemination plans, including Easy Read summaries, short videos, and accessible newsletters.

## 3.3 Project Design

Our previous work co-designing the GeneEQUAL Educational Toolkit in the state of New South Wales with people with intellectual disability[8] informed the design of this current study. Our rationale and assumptions in designing this program were based on this previous successful work.

This three-phase qualitative study progresses from understanding to action to implementation (Figure 2). Each phase builds upon previous findings while maintaining the



For peer review only - http://bmjopen.bmj.com/site/about/guidelines.xhtml

For peer review only

continuous involvement of people with intellectual disability. The project will run from the time of Phase 1 ethics approval until October 2028, with data collection anticipated to occur shortly after the program starts until January 2028.

### 3.3.1 Phase 1: Exploring Barriers and Enablers to Inclusive Genetic Healthcare

We will recruit four groups totalling approximately 100 people to participate in qualitative research to help us explore in depth barriers and enablers to inclusive, person-centred and respectful genetic healthcare. The groups will be: 1. People with intellectual disability with genetic testing and/or counselling experience, 2. People with intellectual disability without genetic testing and/or counselling experience, 3. Family members/support people, and 4. Healthcare professionals. We will use convenience sampling but will strive to maximise diversity across age, gender, ethnicity, and location.

To ensure all participants can contribute, we will offer flexible and accessible ways to participate, including interviews or focus groups (online or in person), in person arts-based methods (Photo-voice, body-mapping)[39,40], and yarning circles for Aboriginal and Torres Strait Islander participants. Yarning in groups (i.e. a yarning circle) or in one-on-one conversations (i.e. "a yarn") are a recognised qualitative research method and offer an alternative for Aboriginal and/or Torres Strait Islander people who prefer these.[41,42] We estimate that interviews and focus groups will last 30-60 minutes; they will be audio-recorded (with informed consent) and transcribed. Interview and focus group protocols will be co-designed through an iterative process, drawing on literature review findings, previous GeneEQUAL studies, and collaborative input from research team experts including researchers with intellectual disability, family members/support people, and healthcare professionals. This ensures protocols are accessible to people with intellectual disability or



For peer review only - http://bmjopen.bmj.com/site/about/guidelines.xhtml

other diverse needs, academically grounded and experientially relevant. The full interview and focus group protocols are available from the corresponding author.

We will use inductive content analysis, a qualitative method well-suited to exploratory research.[43] The analysis will follow a five-step process: (1) familiarisation through thorough reading of all transcripts, (2) open coding to identify units of meaning, (3) category development, where categories and subcategories are developed in parallel through iterative refinement of the codes, (4) comparison and refinement of all categories and subcategories, and (5) thematic abstraction to develop overarching themes and subthemes that synthesise the categories that can answer our research questions.[44]

Data from arts-based methods will be captured through multiple channels. For photo-voice, participants will take or select photographs representing their experiences, and discuss the meaning and significance of each photograph with a researcher. These discussions will be audio-recorded and transcribed alongside the photographs themselves. For body-mapping, participants' visual representations will be documented through high-resolution photography and accompanied by participant narratives explaining their choices, which will also be audio-recorded and transcribed. The visual data (photographs and body maps) will be analysed alongside the transcripts, with attention to both the visual content and participants' interpretations of their own work.

The yarning circles and individual yarns will be audio-recorded (with informed consent) and transcribed. Yarning will be led by Indigenous researchers and will follow culturally appropriate protocols, including allowing conversations to flow naturally rather than adhering to a rigid interview schedule. All transcribed data from arts-based methods and yarning will be incorporated into the inductive content analysis described above, alongside data from interviews and focus groups, ensuring that all forms of data contribute equally to the findings.



For peer review only - http://bmjopen.bmj.com/site/about/guidelines.xhtml

To enhance rigour, multiple researchers will code independently, followed by meetings to reach consensus on data organisation.[45] Field notes and mind-maps will be used to prompt data analysis.[46] Regular team discussions and participant validation will ensure findings remain grounded in participants' experiences.

### 3.3.2 Phase 2: Co-designing Guiding Principles for Inclusive Genetic Healthcare

The Guiding Principles Development Group (GPDG) will be guided by Phase 1 findings. Meetings will be accessible, and materials will be developed in Easy Read to ensure people with intellectual disability can contribute fully.

Development and evaluation will follow the Appraisal Guidelines for Research and Evaluation (AGREE) II-compliant processes,[47] using the Grading of Recommendations, Assessment, Development and Evaluation (GRADE) framework.[48] The draft principles will be evaluated by expert review and consultations. When analysing the collected feedback, we will use researcher triangulation (where several team members independently review and code the same data) and member checking (where participants confirm whether our interpretations of the data accurately reflect their perspectives) to ensure consistency and reliability.

To ensure the national Guiding Principles are a 'living document', regular biannual meetings of the GPDG will be held throughout the project to update the Guiding Principles, guided by our Advisory Board and CEGs.

### 3.3.3 Phase 3: Co-develop a Suite of Resources for Implementation of Best Practice

Using our pilot study findings and stakeholder input, we will co-develop a suite of resources including educational materials such as videos, Easy Read social stories, and fact



For peer review only - http://bmjopen.bmj.com/site/about/guidelines.xhtml

sheets; communication tools for people with high support needs; and training resources for genetic healthcare professionals. CEG workshops will guide our decisions about content, media, and design preferences. These workshops and associated materials will be in a format that is accessible to people with intellectual disability.

We will evaluate the impact of the resources on people with intellectual disability via focus groups conducted during the co-design process and accessible interviews conducted once the resources are completed. Focus groups will be recorded, transcribed verbatim, and analysed using inductive content analysis.

The impact of the resources on families/carers will be evaluated through online surveys and interviews after they have interacted with the resources. The survey incorporates questions from the Patient Activation Measure (PAM) and Genomics Outcome Scale (GOS) items,[49,50] and may be updated during the co-design process in line with identified needs and priorities. These measures were chosen because they will capture the impact of the resources on each individual's readiness and ability to take an active role in their family member's health and healthcare (PAM), and the personal and psychosocial impact of the resources (GOS).

Post-pre surveys similar to those used to assess the toolkit developed as part of our pilot study and based on the Capability Opportunity Motivation and Behaviour (COM-B) framework will explore whether healthcare professionals' capability, motivation and opportunity to apply best practice have changed after using the resources.[8]

The resources will be rolled out nationally, facilitated by the Investigators and Advisory Board, with the help of their wide and diverse networks. We will conduct an Intervention Scalability Assessment[51] with 30-40 stakeholders at inclusive workshops across all Australian States and Territories to identify factors that help or hinder implementation and



For peer review only - http://bmjopen.bmj.com/site/about/guidelines.xhtml

use of the resources. The implementation strategy will be revised in line with these findings, as needed.

## 3.4 Sample Size and Data Saturation

Our sample size is based on established principles for qualitative research.[52] For Phase 1 data collection, we will aim for approximately 100 participants across the four stakeholder groups, with around 25 people per group. Recent systematic reviews show that qualitative research consistently reaches a point defined as saturation within 9-17 interviews or 4-8 focus group discussions, particularly for studies with similar populations and clearly defined objectives.[53,54] Data saturation, the point when new data become redundant and repeat what was previously expressed, is particularly relevant to inductive content analytic approaches.[43] Our pilot study with 15 participants with intellectual disability reached data saturation, which supports our proposed sample size.[34] Applying these principles to data collection: if new data repeat what was expressed in previous data, with no new content categories or themes emerging after 3 additional interviews, we will stop recruitment for that group. However, if new themes or content categories are identified, we will extend recruitment up to 35 per group to ensure comprehensive coverage.[53]

For Phase 2, each GPDG will have 4 experts, which will be sufficient to provide a broad perspective and expertise in the development of the Guiding Principles. The Advisory Board has 48 members.

For Phase 3, each CEG involved in developing resources will comprise at least 6 participants (18 in total). This aligns with focus group methodology to attain diverse community feedback while ensuring a comfortable group environment.[55,56] The multi-stakeholder evaluation of the co-produced resources will recruit a minimum of 25 participants



For peer review only - http://bmjopen.bmj.com/site/about/guidelines.xhtml

per stakeholder group (matching Phase 1 cohorts) to obtain diverse community feedback, reflecting common qualitative research practice.

## 4 ETHICS AND DISSEMINATION

### 4.1 Ethics Approvals

Ethics approval was granted by the UNSW Sydney Human Research Ethics Committee (HC230353) and the Australian Institute of Aboriginal and Torres Strait Islander Studies (AIATSIS; REC-0284). Our approach respects Indigenous data sovereignty principles, with Indigenous leadership guiding how information is collected, interpreted, and shared.

### 4.2 Informed consent

We use a voluntary written informed consent process for all participants, including those with intellectual disability. This approach is based on extensive feedback from people with intellectual disability, that assuming incapacity to consent is offensive and risks triggering post-traumatic stress from previous experiences where they were required to prove their capacity.[8] The project team includes co-researchers with intellectual disability, reflecting our commitment to people with intellectual disability being active participants in research decisions. We will use a continuous consent process and include reasonable adjustments such as extended time, and Easy Read and plain English participant information sheets and consent forms as appropriate. We will use the Teach-Back method to ensure understanding, where participants explain information they have been given in their own words, researchers clarify any misunderstandings, and this process continues until comprehension is achieved.[57] Participants will be encouraged to bring someone they trust to interviews, and will be assured that they can withdraw at any time without affecting their relationship with researchers.



For peer review only - http://bmjopen.bmj.com/site/about/guidelines.xhtml

### 4.3 Data Management

Data will be audio-recorded and transcribed by the research team, with all identifiers removed. Pseudonyms will replace names and the master de-identification list will be stored separately from anonymised data on secure, password-protected systems accessible only to team members.

### 4.4. Safety Monitoring

The research team is experienced in working with people with intellectual disability. We recognise there are potential harms associated with genetic healthcare in people with intellectual disability, such as uncomfortable or worrying resonances with historical eugenics programs, or the possibility that individuals may perceive themselves as 'faulty' if a genetic variant is responsible for their intellectual disability.

Although no concerns were identified during our pilot project, to minimise the risk of potential harm, interviews will be conducted within a trauma-informed framework by a co-researcher with intellectual disability and/or a researcher with many years' experience working with people with intellectual disability, and a language guide will be applied across the project to ensure emotive or negative language is not used. A safety protocol is in place to manage any participant distress, including pausing or stopping the interview/focus group, debriefing after the session, and arranging appropriate support if needed through family members, support workers, or general practitioners.

We will implement measures to support self-care and researcher safety and well-being, to build researcher resilience and prevent vicarious trauma.[58]

For peer review only

### 4.4 Dissemination Plan

All materials will be available on our accessible website (www.GeneEQUAL.com). Findings will be disseminated through peer-reviewed publications, conference presentations, health professional and self-advocacy websites, and social media. Participants will receive accessible summaries of findings via email or video, with researchers available to answer questions as necessary.

## 5 DISCUSSION

The GeneEQUAL project addresses a critical gap in healthcare equity by developing the world's first inclusive, person-centred genetic healthcare model for people with intellectual disability. Our pilot research revealed that people with intellectual disability frequently feel unsupported in genetic healthcare, with numerous missed opportunities for obtaining genetic diagnoses that could inform practical healthcare plans. This protocol outlines our approach to transforming this situation through rigorous co-production methodologies.

The strength of our approach lies in genuine co-production, where people with intellectual disability contribute as active researchers rather than passive subjects. Our three-phase design deliberately moves from understanding barriers, to developing and then implementing solutions. Using different data collection methods (including arts-based approaches [39,59] and yarning circles[60]) allows people to participate in line with their communication preferences and ways of being, knowing and doing.[61] The resulting Guiding Principles will be distinctive in having emerged directly from lived experience rather than from professional assumptions of what people need.

We anticipate several practical outcomes. For people with intellectual disability, the resources will support meaningful participation in healthcare decisions. For their families and support people it will enable accessible and strengths-based communication about genetics.



For peer review only - http://bmjopen.bmj.com/site/about/guidelines.xhtml

For healthcare professionals, the project will provide evidence-based approaches to inclusive healthcare. For healthcare systems, examining how to implement a more inclusive model of genetic healthcare will identify pathways for scaling these approaches nationally.

A central challenge is that meaningful co-production is resource-intensive and requires sustained commitment from all stakeholders. Our layered engagement structure aims to address this by distributing responsibility across the CEGs. Additionally, the heterogeneity of intellectual disability means resources must be adaptable to different support needs and cultural contexts, which our emphasis on multimodal resources and iterative testing aims to address.

While focused on Australia, this work has broad international relevance. The exclusion of people with intellectual disability from advances in genetic healthcare is a global phenomenon. Our methodological approach offers a template that could be adapted to other healthcare systems and may also inform co-production approaches in other clinical fields where people with intellectual disability face similar barriers to healthcare participation.

In the long term**,** this research will foster a paradigm shift from health research that is done *on* people with intellectual disability to that which *is led by* people with intellectual disability. It can also serve as a model for other areas of health, given the urgent need for radical improvement in all healthcare for people with intellectual disability. The mentoring of co-researchers through our program will also grow the next generation of researchers with intellectual disability.



For peer review only - http://bmjopen.bmj.com/site/about/guidelines.xhtml

## Authors contributions

IS, EEP, JLR and JLS conceived the idea of the project. IS, JLR, EEP, JLS, GP, ET, SB, SS and JM developed the study concept and initiated the project intervention. IS, MS and EEP led the development of an in-depth protocol. EEP, IS, MT and NR wrote the manuscript. All authors reviewed and provided input into the manuscript and read and approved the final version.

EEP is the guarantor.

**Funding statement**

This work was supported by the Australian National Health & Medical Research Council (NHMRC) Targeted Call for Research into Improving Health of People with Intellectual disability. Research grant title "GeneEQUAL: equitable and accessible genomic healthcare for people with intellectual disability". NHMRC application ID: 2022/GNT2015753. EEP is supported by a National Health and Medical Research Council investigator grant (2021/GNT2008166)

## Competing interests

The authors declare the following competing interests. IS, JLR, JD, JM, SB, ET, JLS, GP and EEP have received funding to their institutions from the NHMRC. MT, SS, CPM, SH, SH, CK have received funding for research assistance from the NHMRC. JB has received funding for a PhD Scholarship from the NHMRC. KBS has received consultancy fees from Belkin Consulting and payment for honoraria for conference and support for travel from UNIKOM Bandung Indonesia. EEP has received funding to attend meetings from the national peak body for rare diseases, Rare Voices Australia. All other authors have no competing interest to declare.



For peer review only - http://bmjopen.bmj.com/site/about/guidelines.xhtml

**Acknowledgements**

The authors acknowledge the GeneEQUAL Advisory Board (in alphabetical order): Yemima Berman, Donna Best, Aimee Blackam, Jasmine Bojadzieva, Emma Bonser, Tiffany Boughtwood, Marie Brand, Mary Butterworth, Emily Caska, Samantha Cooms, Emily Cukalevski, Rachael Cvejic, Dalal Dawood Baumgartner, Leon Elman, Anna Flynn, Emma Gibson, Sally Heggaton, Falak Helwani, Paul Jarnet, Nick Lennox, Trinity Mahede, Belinda McClaren, Cassandra McDonald, Saras Menon, Cathy Milne, Laura Naing, Mary O'Donovan, Trevor Parmenter, Trupti Prasad, Thulasee Sri Ganeshan, Tracey Szanto, Georgina Schlub, Maree Toombs, Michelle Ward

## REFERENCES

1. American Psychiatric Association. Diagnostic and statistical manual of mental disorders (DSM-5®). https://doi/book/10.1176/appi.books.97808904255962013 2013.
2. Dunn M, Strnadova I, Scully JL, *et al.* Equitable and accessible informed healthcare consent process for people with intellectual disability: a systematic literature review. *BMJ Qual Saf* 2024;33:328-39.
3. Ali A, Scior K, Ratti V, *et al.* Discrimination and other barriers to accessing health care: perspectives of patients with mild and moderate intellectual disability and their carers. *PLoS ONE* 2013;8:e70855.
4. Mimmo L, Harrison R, Hinchcliff R. Patient safety vulnerabilities for children with intellectual disability in hospital: a systematic review and narrative synthesis. *BMJ Paediatr Open* 2018;2:e000201.
5. United Nations. Convention on The Rights of Persons with Disabilities (A/RES/61/106). https://social.desa.un.org/issues/disability/crpd/convention-on-the-rights-of-persons-with-disabilities-crpd. 2006.
6. Molnar C, Strnadova I, Dunn M, *et al.* The need for co-educators to drive a new model of inclusive, person-centred and respectful co-healthcare with people with intellectual disability. *Front Psychiatry* 2024;15:1346423.
7. Brooker KS, De Greef R, Trollor JN, *et al.* Intellectual disability healthcare in Australia: Progress, challenges, and future directions. *Journal of Policy and Practice in Intellectual Disabilities* 2024;21:e12497.



For peer review only - http://bmjopen.bmj.com/site/about/guidelines.xhtml

8. Strnadova I, Dunn M, Molnar C*, et al.* "All doctors should be trained in that": The co-production and mixed-methods evaluation of an educational toolkit to enable safe, high-quality genetic healthcare for people with intellectual disability. *Genet Med* 2025:101371.

9. Trollor J, Srasuebkul P, Xu H, Howlett S. Cause of death and potentially avoidable deaths in Australian adults with intellectual disability using retrospective linked data. *BMJ Open* 2017;7:e013489.

10. Walmsley J. Investigating premature deaths of people with intellectual disabilities: who is protecting whom? *Disabil Soc* 2020;35:1018-23.

11. El Ansari W, Stock C. Is the Health and Wellbeing of University Students Associated with their Academic Performance? Cross Sectional Findings from the United Kingdom. 2010;7:509-27.

12. Bourke J, Nembhard WN, Wong K, Leonard H. Twenty-Five Year Survival of Children with Intellectual Disability in Western Australia. *J Pediatr* 2017;188:232-9 e2.

13. Australian Government Royal Commission into Violence Abuse Neglect and Exploitation of People with Disability. Final Report - Volume 3, Nature and extent of violence, abuse, neglect and exploitation. https://disability.royalcommission.gov.au/publications/final-report-volume-3-nature-and-extent-violence-abuse-neglect-and-exploitation. 2023.

14. Hammarlund M, Granqvist P, Forslund T. Experiences of Interpersonal Trauma Among Parents With Intellectual Disabilities: A Systematic Review. *Trauma Violence Abuse* 2023;24:2843-62.

15. Australian Government Department of Health Disability and Ageing. National Roadmap for Improving the Health of People with Intellectual Disability. https://www.health.gov.au/our-work/national-roadmap-for-improving-the-health-of-people-with-intellectual-disability. 2021.

16. Vissers LE, Gilissen C, Veltman JA. Genetic studies in intellectual disability and related disorders. *Nat Rev Genet* 2016;17:9-18.

17. Srivastava S, Love-Nichols JA, Dies KA*, et al.* Meta-analysis and multidisciplinary consensus statement: exome sequencing is a first-tier clinical diagnostic test for individuals with neurodevelopmental disorders. *Genetics in Medicine* 2019;21:2413-21.

18. Miller DT, Adam MP, Aradhya S*, et al.* Consensus statement: chromosomal microarray is a first-tier clinical diagnostic test for individuals with developmental disabilities or congenital anomalies. *Am J Hum Genet* 2010;86:749-64.

19. Tan TY, Dillon OJ, Stark Z*, et al.* Diagnostic Impact and Cost-effectiveness of Whole-Exome Sequencing for Ambulant Children With Suspected Monogenic Conditions. *JAMA Pediatr* 2017;171:855-62.

20. Yang Y, Muzny DM, Reid JG*, et al.* Clinical whole-exome sequencing for the diagnosis of mendelian disorders. *N Engl J Med* 2013;369:1502-11.



For peer review only - http://bmjopen.bmj.com/site/about/guidelines.xhtml

21. Lee H, Deignan JL, Dorrani N, *et al.* Clinical exome sequencing for genetic identification of rare Mendelian disorders. *JAMA* 2014;312:1880-7.

22. Yang Y, Muzny DM, Xia F, *et al.* Molecular findings among patients referred for clinical whole-exome sequencing. *Jama* 2014;312:1870-9.

23. Retterer K, Juusola J, Cho MT, *et al.* Clinical application of whole-exome sequencing across clinical indications. *Genet Med* 2016;18:696-704.

24. van Karnebeek CDM, Houben RFA, Lafek M, *et al.* The treatable intellectual disability APP www.treatable-id.org: a digital tool to enhance diagnosis & care for rare diseases. *Orphanet J Rare Dis* 2012;7:47.

25. Coulter ME, Miller DT, Harris DJ, *et al.* Chromosomal microarray testing influences medical management. *Genetics in Medicine* 2011;13:770-6.

26. Savatt JM, Myers SM. Genetic testing in neurodevelopmental disorders. *Front Pediatr* 2021;9:526779.

27. Kohler JN, Turbitt E, Biesecker BB. Personal utility in genomic testing: a systematic literature review. *Eur J Hum Genet* 2017;25:662-8.

28. Strnadová I, Dowse L, Garcia-Lee B. Doing Research Inclusively: Co-Production in Action. DIIU UNSW Sydney. https://www.disabilityinnovation.unsw.edu.au/inclusive-research/research-resources 2022.

29. Kotwal H, Fleming J, Barlow-Stewart K, *et al.* Pre-genetics clinic resource evaluation for adults with intellectual disability: The pre-genetics clinic aid. *J Genet Couns* 2020;29:668-77.

30. Australian Institute Of Health and Welfare. Intellectual disability in Australia's Aboriginal and Torres Strait Islander peoples. *J Intellect Dev Disabil* 2007;32:222-5.

31. Bourke J, Sanders R, Jones J, *et al.* Intellectual disability and autism prevalence in Western Australia: impact of the NDIS. *Front Psychiatry* 2024;15:1359505.

32. Hamilton SL, Maslen S, Watkins R, *et al.* 'That thing in his head': Aboriginal and non-Aboriginal Australian caregiver responses to neurodevelopmental disability diagnoses. *Sociol Health Illn* 2020;42:1581-96.

33. Australian Government Department of Health Disability and Ageing. Genomics Australia. https://www.health.gov.au/our-work/genomics-australia.

34. Strnadova I, Loblinzk J, Scully JL, *et al.* "I am not a number!" Opinions and preferences of people with intellectual disability about genetic healthcare. *Eur J Hum Genet* 2023;31:1057-65.

35. Bogna F, Raineri A, Dell G. Critical realism and constructivism: merging research paradigms for a deeper qualitative study. *Qualitative Research in Organizations and Management: An International Journal* 2020;15:461-84.

36. Berryman DR. Ontology, Epistemology, Methodology, and Methods: Information for Librarian Researchers. *Med Ref Serv Q* 2019;38:271-9.



For peer review only - http://bmjopen.bmj.com/site/about/guidelines.xhtml

37. Hathcoat JD, Meixner C, Nicholas MC. Ontology and Epistemology. In: Liamputtong P, editor. Handbook of Research Methods in Health Social Sciences. Singapore: Springer Singapore; 2019. p. 99-116.

38. Spencer R, Pryce JM, Walsh J. Philosophical Approaches to Qualitative Research. In: Leavy P, editor. The Oxford Handbook of Qualitative Research: Oxford University Press; 2020. p. 112-42.

39. Danker J, Strnadova I, Cumming TM. Picture my well-being: Listening to the voices of students with autism spectrum disorder. *Res Dev Disabil* 2019;89:130-40.

40. Leavy P. Method meets art: Arts-based research practice, 2nd ed. New York, NY, US: Guilford Press; 2015. xxi, 328-xxi, p.

41. Kennedy M, Maddox R, Booth K*, et al.* Decolonising qualitative research with respectful, reciprocal, and responsible research practice: a narrative review of the application of Yarning method in qualitative Aboriginal and Torres Strait Islander health research. *International Journal for Equity in Health* 2022;21:134.

42. Lewis T, Hill AE, Bond C, Nelson A. Yarning. *Journal of Clinical Practice in Speech-Language Pathology* 2017;19:14-8.

43. Saunders B, Sim J, Kingstone T*, et al.* Saturation in qualitative research: exploring its conceptualization and operationalization. *Qual Quant* 2018;52:1893-907.

44. Vears D, Gillam L. Inductive content analysis: A guide for beginning qualitative researchers. *Focus on Health Professional Education: A Multi-Professional Journal* 2022;23:111-27.

45. Lovrić R, Farčić N, Mikšić Š, Včev A. Studying During the COVID-19 Pandemic: A Qualitative Inductive Content Analysis of Nursing Students' Perceptions and Experiences. *Education Sciences* 2020;10:188.

46. Cascio MA, Racine E. Person-oriented research ethics: integrating relational and everyday ethics in research. *Account Res* 2018;25:170-97.

47. Brouwers MC, Kho ME, Browman GP*, et al.* AGREE II: advancing guideline development, reporting and evaluation in health care. *CMAJ* 2010;182:E839-42.

48. Prasad M. Introduction to the GRADE tool for rating certainty in evidence and recommendations. *Clinical Epidemiology and Global Health* 2024;25:101484.

49. Hibbard JH, Stockard J, Mahoney ER, Tusler M. Development of the Patient Activation Measure (PAM): Conceptualizing and Measuring Activation in Patients and Consumers. *Health Services Research* 2004;39:1005-26.

50. Grant PE, Pampaka M, Payne K*, et al.* Developing a short-form of the Genetic Counselling Outcome Scale: The Genomics Outcome Scale. *Eur J Med Genet* 2019;62:324-34.

51. Milat A, Lee K, Conte K*, et al.* Intervention Scalability Assessment Tool: A decision support tool for health policy makers and implementers. *Health Res Policy Syst* 2020;18:1.



For peer review only - http://bmjopen.bmj.com/site/about/guidelines.xhtml

52. Braun V, Clarke V. To saturate or not to saturate? Questioning data saturation as a useful concept for thematic analysis and sample-size rationales. *Qualitative Research in Sport, Exercise and Health* 2021;13:201-16.

53. Hennink M, Kaiser BN. Sample sizes for saturation in qualitative research: A systematic review of empirical tests. *Soc Sci Med* 2022;292:114523.

54. Wutich A, Beresford M, Bernard HR. Sample Sizes for 10 Types of Qualitative Data Analysis: An Integrative Review, Empirical Guidance, and Next Steps. *International Journal of Qualitative Methods* 2024;23:16094069241296206.

55. Velez CV, Campez-Pardo M, Canovas JM*, et al.* An Iteratively Adapted Transdiagnostic Prevention Program for Diverse High School Settings (U-PEACE): Protocol for a Randomized Controlled Trial. *JMIR Res Protoc* 2025;14:e74080.

56. Burns C, Kilkenny MF, Purvis T*, et al.* Processes Underpinning Successful Co-Design: Lessons From a Digital Health Project. *Health Expect* 2025;28:e70272.

57. Talevski J, Wong Shee A, Rasmussen B*, et al.* Teach-back: A systematic review of implementation and impacts. *PLoS ONE* 2020;15:e0231350.

58. Smith E, Pooley JA, Holmes L*, et al.* Vicarious Trauma: Exploring the Experiences of Qualitative Researchers Who Study Traumatized Populations. *Disaster Med Public Health Prep* 2021;17:e69.

59. Danker J, Strnadová I, Cumming TM. Engaging Students With Autism Spectrum Disorder in Research Through Participant-Driven Photo-Elicitation Research Technique. *Australasian Journal of Special Education* 2017;41:35-50.

60. Christensen K. Yarning as decolonising practice. *Int J Narrat Ther Community Work* 2022:1-8.

61. Bessarab D. Changing How and What We Do: The Significance of Embedding Aboriginal and Torres Strait Islander Ways of Knowing, Being, and Doing in Social Work Education and Practice. *Australian Social Work* 2015;68:1-4.



For peer review only - http://bmjopen.bmj.com/site/about/guidelines.xhtml

**FIGURES**

**Figure 1.** Project engagement structure

**Figure 2.** Project phases

For peer review only



For peer review only - http://bmjopen.bmj.com/site/about/guidelines.xhtml

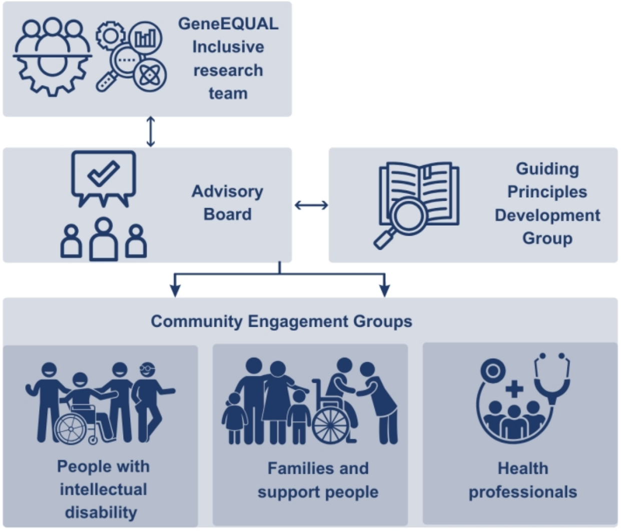


Figure 1. Project engagement structure

758x536mm (67 x 67 DPI)

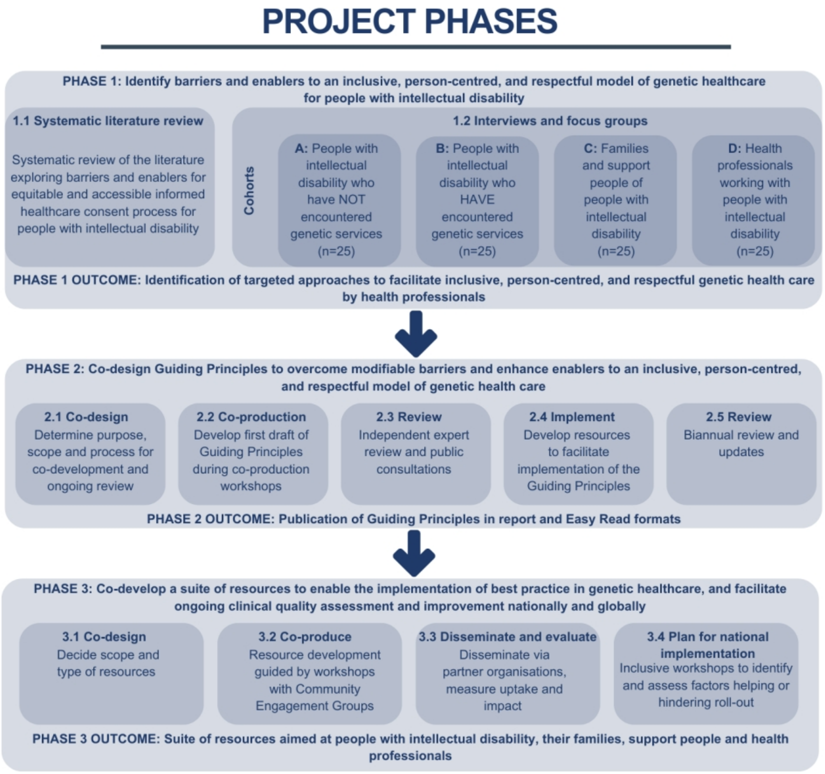


Figure 2. Project phases

536x758mm (67 x 67 DPI)

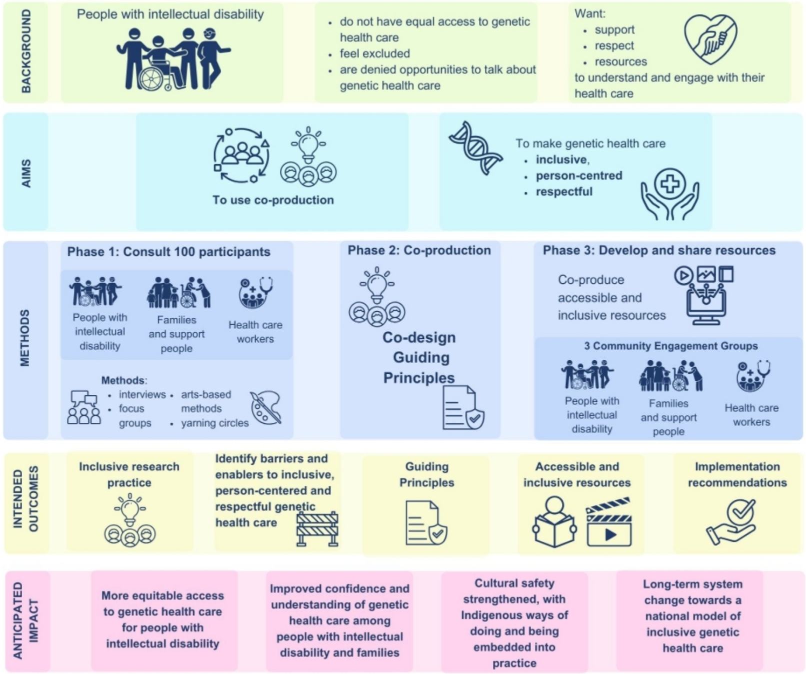


297x210mm (171 x 171 DPI)

# SUPPLEMENTAL MATERIAL

## Easy Read Summary

## Making Genetic Health Care Better For People With Intellectual Disability

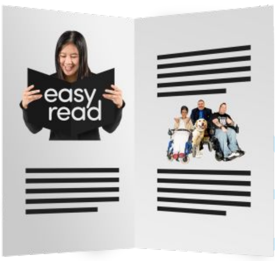


This booklet is to explain a **research study**

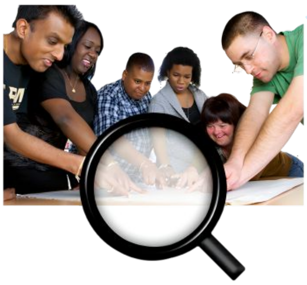

A **research study** is something we do

- when we want to find information

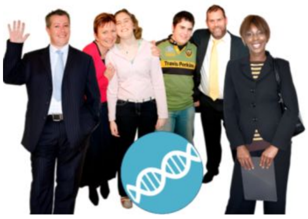

This research study will be about

- people with intellectual disability and
- genetic health care

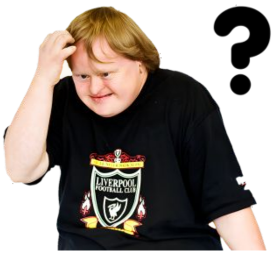

Some things in the booklet can be hard to understand

For peer review only



For peer review only - http://bmjopen.bmj.com/site/about/guidelines.xhtml

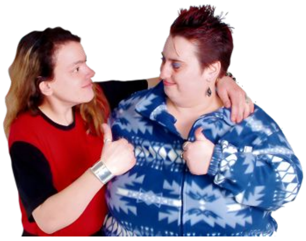

You can read it with someone you trust

For example, a family member, friend, or health care worker

## What is genetic health care?

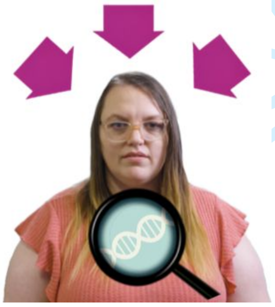

Genetic health care is for people who

- have a **genetic condition**
- might have a genetic condition
- want to know more about genetic conditions and tests

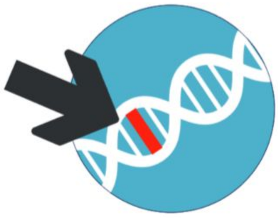

A **genetic condition** happens if a gene changes

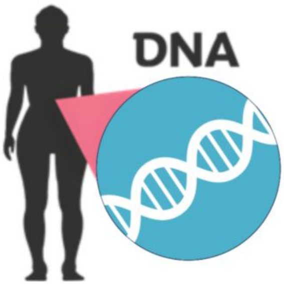


Genes are the things our body

- that make us the way we are

For peer review only

Genetic health care is important for all people

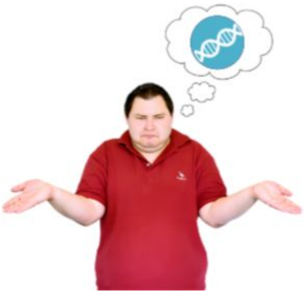

This is because we may have a genetic condition

- and we do not know about it

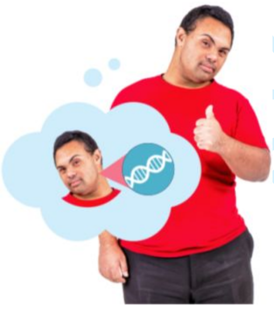

If we know about our genetic condition we can

- understand things about us
- have better health treatment
- prepare for future problems

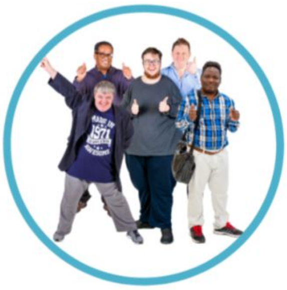

It can also help us to

- meet people with same genetic condition
- make decisions
- have a good life

## Why are we doing this study?

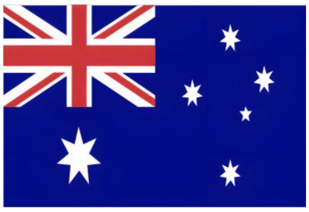

In 2007 Australia signed a document called

- United Nations Convention on the Rights of People with Disabilities



For peer review only - http://bmjopen.bmj.com/site/about/guidelines.xhtml

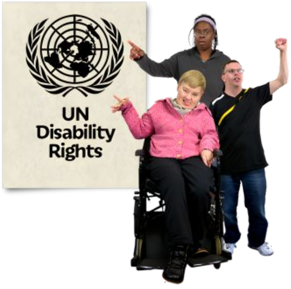


Australia wants people with disability

- to have the same rights as everyone else

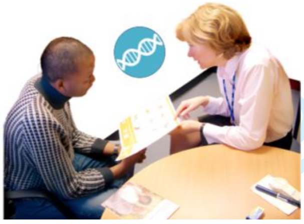

Having the same rights includes

- having the best possible genetic health care

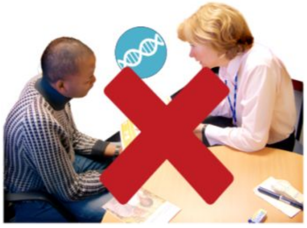

But people with intellectual disability do not have

- the best genetic health care

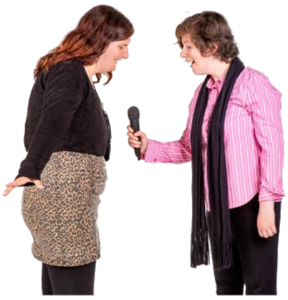

In our previous study, we asked people with intellectual disability

- about their experience with genetic health care



For peer review only - http://bmjopen.bmj.com/site/about/guidelines.xhtml

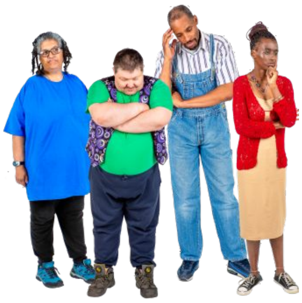

They told us they want support

They do not feel included and feel sad

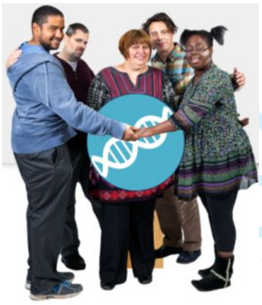

People want to

- understand genetic health care
- be treated with respect
- have the right resources

## What do we want to do in this study?

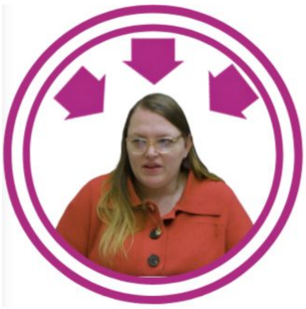

We want to make genetic health care

- **inclusive**
- **person centred**
- **respectful**

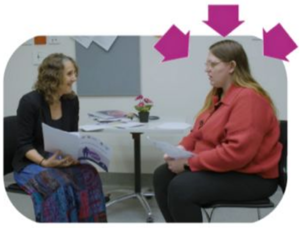

**Inclusive, person centred and respectful**

means that health care workers

- listen to you
- treat you well
- give you time to understand
- help you make the right decision



For peer review only - http://bmjopen.bmj.com/site/about/guidelines.xhtml

# How are we going to do it?

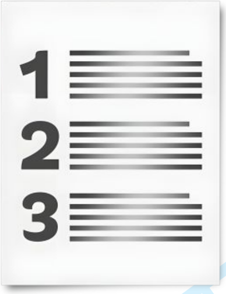

We will do 3 things

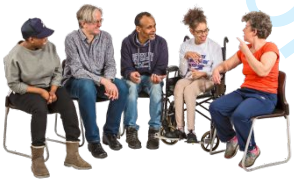

We will ask people about genetic health care

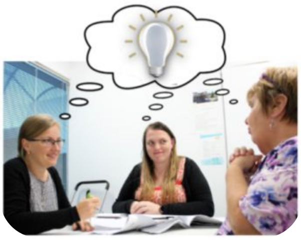

Together we will think about answers

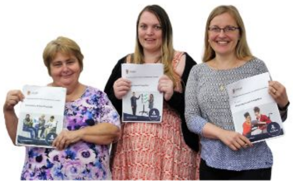

We will share our answers

We will make resources to support health care workers

For peer review only

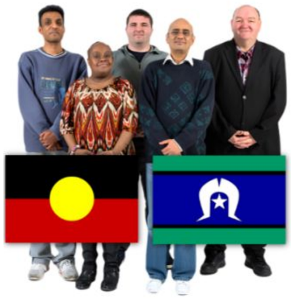

We will work with people with intellectual disability

- who are Aboriginal and Torres Strait Islander

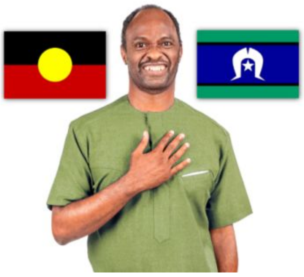

We will make sure to be **culturally safe**

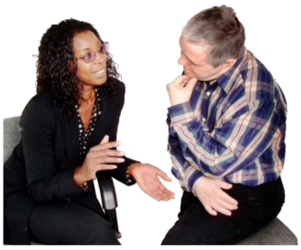

**Culturally safe** means we

- respect the history of people
- listen and ask about their preferences
- help people feel comfortable

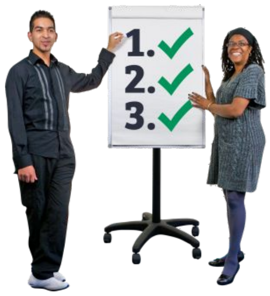


## Our study will happen in 3 steps



For peer review only - http://bmjopen.bmj.com/site/about/guidelines.xhtml

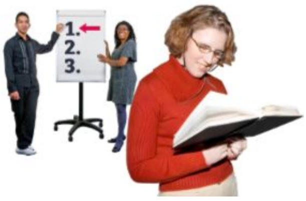


**Step 1**

We will find what other people did

- to improve genetic health care

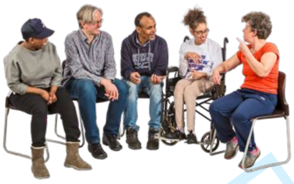

We will talk to 25 Australians from each group

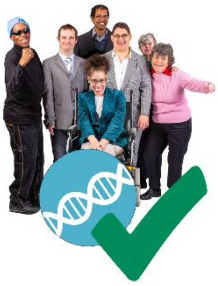

Group 1 will be people with intellectual disability

- who had experience with genetic health care

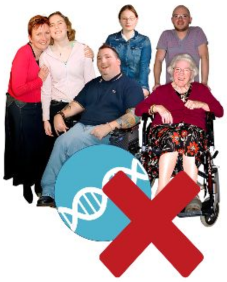

Group 2 will be people with intellectual disability

- who did not have experience with genetic health care

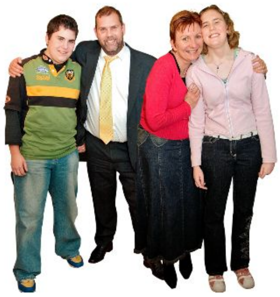

Group 3 will be family members and carers

For peer review only

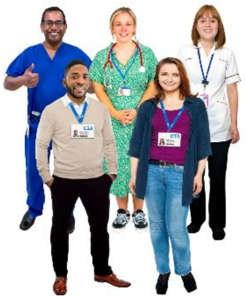

Group 4 will be health care workers

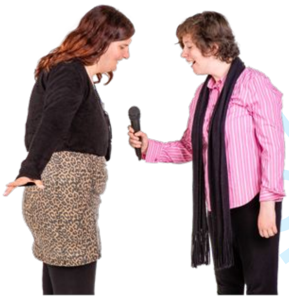

We will use interviews, focus groups and

**yarning circles**

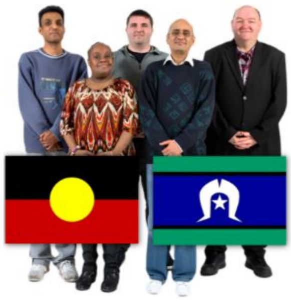

**Yarning circles** are something that Aboriginal and Torres Strait Islander do to

- share stories
- talk to each other

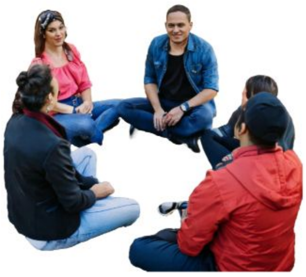

In yarning circles

- people sit in a circle
- everyone gets a turn to speak

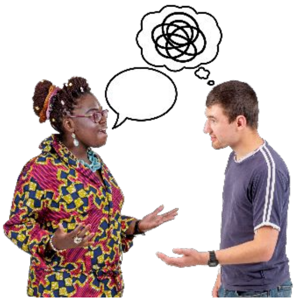

We will also use other things

- to support people to share their ideas with us



For peer review only - http://bmjopen.bmj.com/site/about/guidelines.xhtml

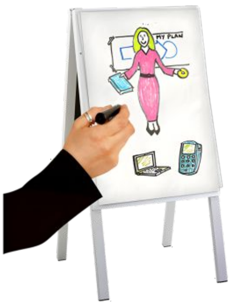

People can make body maps to tell us what they think

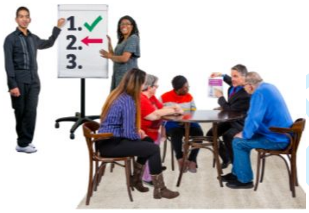


**Step 2**

We will

- talk about what people told us
- think about answers and ideas

**Representatives** of

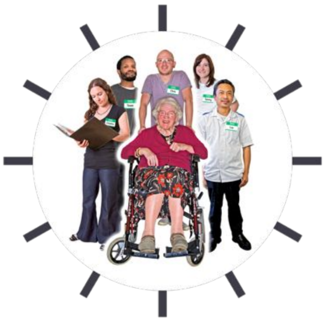

- people with intellectual disability
- their families and carers
- health care workers

will be part of Step 2

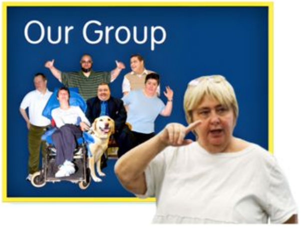


**Representatives** are people

- who speak for their group
- share what their group thinks
- make sure their needs are heard

We will also ask experts in intellectual disability

For peer review only

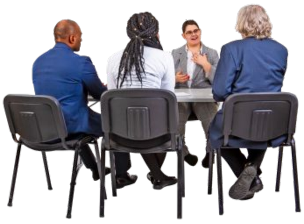

- what they think about our answers and ideas

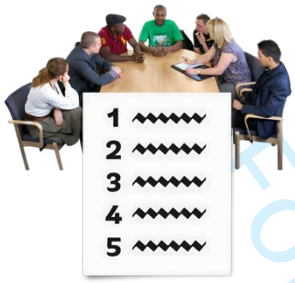


We will then write **Guiding Principles** to support health care workers

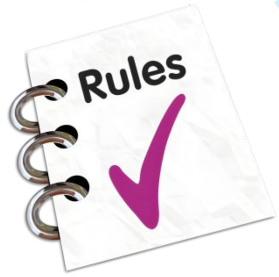


**Guiding Principles** are similar to rules

For peer review only

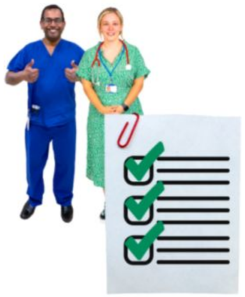

They will tell health care workers

- what is the right thing to do

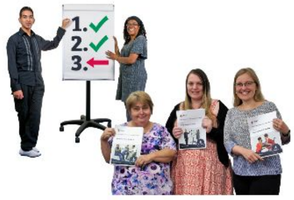


**Step 3**

We will make and share resources

- to help make genetic health care better



For peer review only - http://bmjopen.bmj.com/site/about/guidelines.xhtml

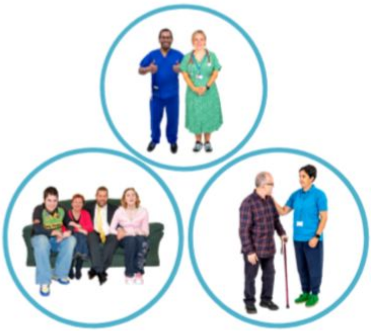

These resources will be for

- people with intellectual disability
- their families and carers
- health care workers

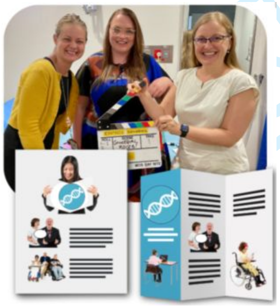

Examples of resources are

- videos
- Easy Read materials
- social stories

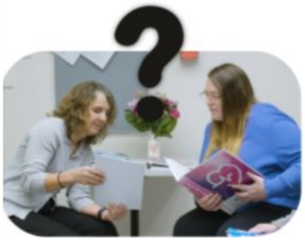

We will also check if

- people are using the resources
- the resources help them

## Doing things the right way

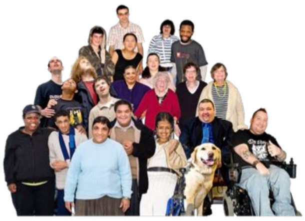

A lot of people will be involved in this research study



For peer review only - http://bmjopen.bmj.com/site/about/guidelines.xhtml

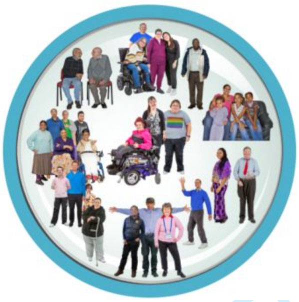

There will be

- participants
- co-researchers
- representatives
- researchers
- families
- health care workers

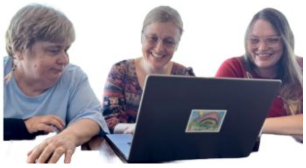

Some people with intellectual disability are co-researchers in our study

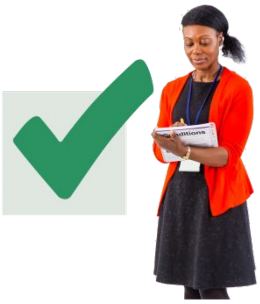

Everyone in our study needs to be safe

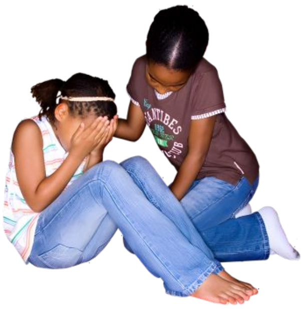

Some people with intellectual disability

- had very bad experiences in their life



For peer review only - http://bmjopen.bmj.com/site/about/guidelines.xhtml

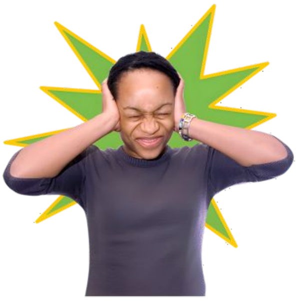

Talking about genetic health care can make them upset

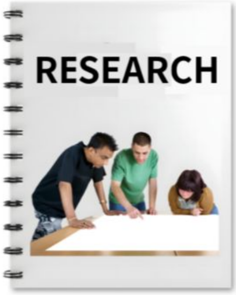


That is why in this study we use a **trauma informed approach**

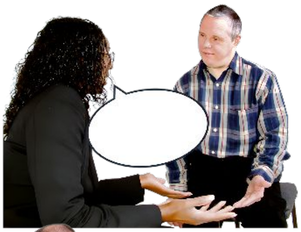

**Trauma informed approach** is about people feeling safe and respected

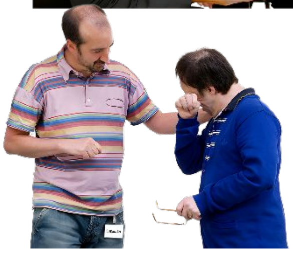

We support people if they need to

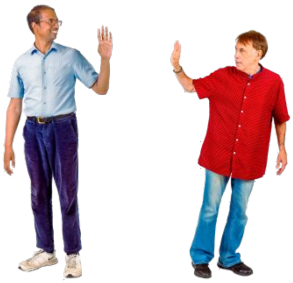

People can also leave our study if they want



For peer review only - http://bmjopen.bmj.com/site/about/guidelines.xhtml

# Who is doing this research study?

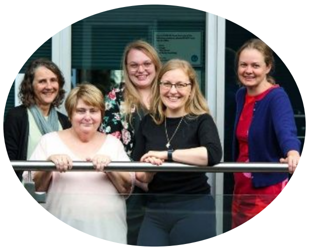

The **GeneEQUAL team** is doing this study

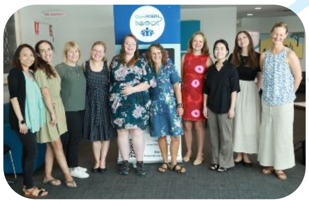

The team includes people

- with intellectual disability
- with other disability
- academic researchers
- health care workers
- teachers

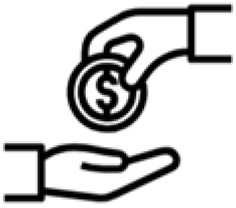

We got funding from the Australian Federal Government (NHMRC)

# Where will I see the results?

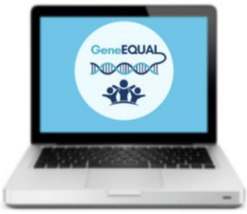


Everything will be available on our website

www.geneequal.com



For peer review only - http://bmjopen.bmj.com/site/about/guidelines.xhtml

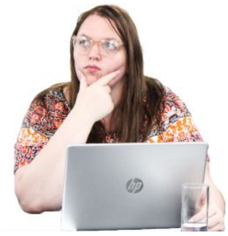

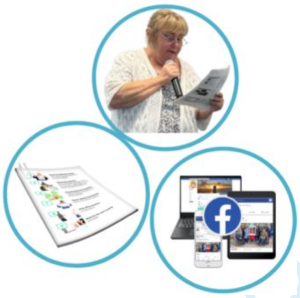

We will also

- write articles
- go to conferences
- publish on other websites
- share in social media

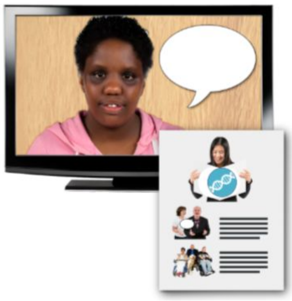

Participants will get our findings in Easy Read and videos

## About this booklet

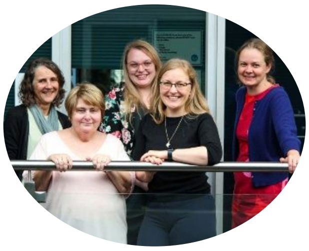

The GeneEQUAL team made this booklet

made with photosymbols®
Photosymbols®
Professional Licence 708948325

It was made using Photosymbols and Adobe



For peer review only

For peer review only - http://bmjopen.bmj.com/site/about/guidelines.xhtml

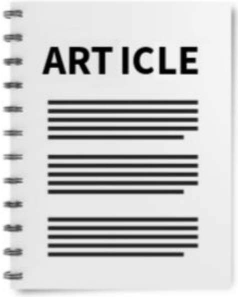


The full article is called:

Developing inclusive genetic healthcare for people with intellectual disability: A co-production research protocol

For peer review only



For peer review only - http://bmjopen.bmj.com/site/about/guidelines.xhtml
